# The adoption of non-pharmaceutical interventions and the role of digital infrastructure during the COVID-19 pandemic in Colombia, Ecuador, and El Salvador

**DOI:** 10.1140/epjds/s13688-023-00395-5

**Published:** 2023-06-06

**Authors:** Nicolò Gozzi, Niccolò Comini, Nicola Perra

**Affiliations:** 1 Networks and Urban Systems Centre, University of Greenwich, London, UK; 2 ISI Foundation, Turin, Italy; 3 The World Bank, Washington, DC USA; 4 School of Mathematical Sciences, Queen Mary University of London, London, UK

**Keywords:** COVID-19, Digital infrastructure, Non-pharmaceutical interventions

## Abstract

Adherence to the non-pharmaceutical interventions (NPIs) put in place to mitigate the spreading of infectious diseases is a multifaceted problem. Several factors, including socio-demographic and socio-economic attributes, can influence the perceived susceptibility and risk which are known to affect behavior. Furthermore, the adoption of NPIs is dependent upon the barriers, real or perceived, associated with their implementation. Here, we study the determinants of NPIs adherence during the first wave of the COVID-19 Pandemic in Colombia, Ecuador, and El Salvador. Analyses are performed at the level of municipalities and include socio-economic, socio-demographic, and epidemiological indicators. Furthermore, by leveraging a unique dataset comprising tens of millions of internet Speedtest® measurements from Ookla®, we investigate the quality of the digital infrastructure as a possible barrier to adoption. We use mobility changes provided by Meta as a proxy of adherence to NPIs and find a significant correlation between mobility drops and digital infrastructure quality. The relationship remains significant after controlling for several factors. This finding suggests that municipalities with better internet connectivity were able to afford higher mobility reductions. We also find that mobility reductions were more pronounced in larger, denser, and wealthier municipalities.

**Supplementary Information:**

The online version contains supplementary material available at 10.1140/epjds/s13688-023-00395-5.

## Introduction

Stay-at-home mandates, travel bans, face masks, curfews, remote working, and closure of non-essential shops are just some examples of the non-pharmaceutical interventions (NPIs) implemented worldwide to contain the spread of SARS-CoV-2 [1–5]. Though extremely successful public health measures, NPIs are associated with high socio-economic costs, might bring economic activities to a halt, and disrupt social life [6, 7].

The literature on the subject suggests that compliance with such measures is a multi-faced problem driven by individual and societal factors. Socio-demographic (e.g., age, gender, educational attainment, country of residence, population density, age pyramid), and epidemiological (e.g., number of cases, deaths, and vaccination rates) indicators play an important role in adherence. They modulate the perceived risk, severity, and susceptibility to the threat and can ultimately affect individual behaviors [1, 8–10]. Adherence to NPIs strongly correlates also with socio-economic determinants [11–14]. From low to high-income countries, disadvantaged communities are affected by structural inequities and as result had markedly fewer opportunities to adopt NPIs during the acute phases of the Pandemic [13, 15–21]. Indeed, while restrictive measures have disrupted the lives of everyone, the challenges and barriers to adoption faced in implementing them are extremely different across socio-economic strata. Informal jobs and several types of occupations, for example, made it extremely hard to implement many forms of NPIs [1, 22, 23].

Widespread adoption of mobile devices and of the Internet allowed to continue remotely some economic, educational, and social activities [24, 25]. These new tools, while opening unprecedented opportunities for many, constituted new barriers for others. Limited and unequal access to a reliable digital infrastructures can influence teleworking, e-commerce adoption, distance learning, use of telehealth platforms, remote access to financial services, and more in general the ability to carry out activities from home thus limiting travels and mobility [25–31]. Arguably, individuals with better internet connections faced far less obstacles while transitioning to online activities with respect to those with poorer digital connectivity which, whenever possible, may have continued to attend essential activities in person.

However, these factors have not been yet extensively explored via a quantitative data-driven approach. Furthermore, the current literature on the subject, apart from a report focused on social distancing in the US during the early phases of 2020 and its relation with access to high-speed internet [32], is mainly focused on specific contexts such as education and telemedicine probed via surveys [33–38].

Here, we tackle this limitation investigating a wide range of variables possibly affecting the adoption of NPIs in the first COVID-19 wave in one lower-middle-income country (El Salvador) and two upper middle-income countries (Colombia and Ecuador) in Latin America. In March 2020, all three countries implemented a series of measures to control the spread of SARS-CoV-2. According to data from the Oxford COVID-19 Government Response Tracker [39], Ecuador was the first to introduce measures restricting internal movement across regions and cities on March 17, 2020, followed by El Salvador on March 18 and Colombia on March 25. At around the same time, the three countries implemented also stay-at-home orders with exceptions for essential trips, closed schools and non-essential/specific sectors workplaces. We explore socio-demographic (i.e., population size, population density, the fraction of the population above 60), socio-economic (i.e., wealth index, GDP per capita), and epidemiological (i.e., number of reported COVID-19 cases) indicators that might affect risk, severity, and susceptibility perception as well as impose barriers to the adoption of NPIs. Additionally, we investigate the quality of the digital infrastructure as a form of barrier that might affect NPIs adherence. To this end, we leverage a unique dataset containing tens of millions of geolocalized internet Speedtest® results from Ookla® [40] that we use as a proxy for quality of internet connection. Measuring the quality of internet connections using Internet Speedtests® or similar tools has become a widely used method, utilized not only by researchers but also by private companies, governments, and other authorities [41–43]. This approach is particularly valuable in identifying disparities in access to reliable and high-quality internet connections across communities [42, 44, 45].

We characterize NPIs adherence using a publicly available dataset from Meta’s Data for Good program that provides high spatio-temporal resolution information about mobility changes [46]. Aggregated mobility indicators, obtained from digital crumbs we leave while using or simply carrying mobile phones in our pockets, together with ad-hoc surveys, have been used to characterize adherence with NPIs at the population level [1]. The scale of the emergency and the strictness of the measures disrupted such a broad range of activities and behaviors that a simple comparison of aggregated mobility volumes (obtained from mobile phones) with respect to a pre-pandemic baseline shows marked differences and allows to quantify adoption of many types of NPIs.

We focus our analyses at the municipal level on three Latin American countries: Colombia, Ecuador, and El Salvador. Latin America is often regarded as the most unequal region in the world [47, 48]. The profound disparities that afflict the region are also reflected in high rates of infection and deaths observed during the Pandemic [49] as well as in the access, use, and quality of digital tools [50, 51]. Analyses conducted within these countries show large spatial heterogeneities and urban-rural divides in many indicators including digital literacy, skills, and access to broadband [25]. Unfortunately, these points, together with the limited number of studies in this region of the world, make Latin American countries good case studies to investigate and expand our knowledge about NPIs adoption.

We find that, during 2020, NPIs significantly affected mobility in the three countries, causing a maximum drop in movements, from pre-pandemic baselines, of 53% in Colombia and 64% in Ecuador and El Salvador. Using different statistical analyses, we first show that municipalities with access to a better internet connectivity - measured with Speedtest® proxy data - also feature more consistent reductions in mobility. Such association is preserved when controlling for possible confounders. We estimate that, for every 10 Megabits per second (Mbps) increase in average fixed download speed, movement reduction increases by another 13%, 4%, 19% in, respectively, Colombia, Ecuador, and El Salvador. We then adopt a regression approach that, besides digital infrastructure quality, accounts also for many other factors possibly influencing mobility reductions in each municipality. We find that digital infrastructure quality is still a significant predictor of NPIs adoption. Population size, density, and socio-economic status are also associated with higher mobility reductions. As a supplementary analysis, we include also regressors on employment and labor force structure (for Colombia only, due to limited data availability). Even in this case, we find that internet quality remains a significant predictor of NPIs adoption.

Notwithstanding clear progress made, much is still unknown about the adherence to NPIs especially when it comes to the effects of different socio-economic barriers, such as the access to a reliable and high-quality internet connection. We aim to extend the literature on the subject offering a quantitative investigation about the role of digital infrastructure quality, along with many other variables, in NPIs compliance during the first wave of COVID-19 Pandemic in three Latin American countries. Understanding such phenomenon is key to informing policies aimed at increasing the resilience to external shocks as well as equity in communities, cities, and countries. Additionally, our research highlights the possibility of utilizing non-conventional data sources, such as Speedtest®, to examine the compliance to NPIs in countries with limited information availability.

## Results

### Proxies of NPIs adoption

**Figure 1:**
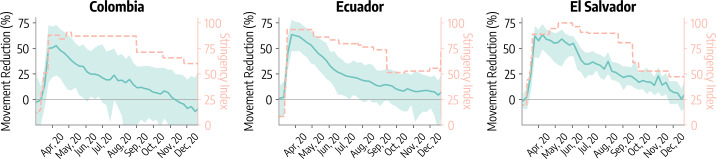
Mobility reductions following the establishment of NPIs in Colombia, Ecuador, and El Salvador. We show the *movement reduction* metric between 2020/03/01 and 2020/12/31 for Colombia, Ecuador, and El Salvador. We show national average (solid line) and the minimum-maximum interval (shaded area) computed over all municipalities in the three countries for which we have data. We also show the stringency index (orange dashed line) of policies implemented to curb COVID-19 spread in the three countries. Horizontal line indicates the pre-pandemic mobility baseline

We characterize NPIs adoption by quantifying how aggregated mobility changed during 2020 in the three countries. To this end, we use the *Movement Range Maps* from Meta’s Data for Good program [46]. This dataset is publicly available and provides the percentage reduction in movement observed with respect to a pre-pandemic baseline (*movement reduction*). Data are available for different municipalities and have temporal resolution of the day. In Fig. 1 we show the weekly evolution of the mobility reduction throughout 2020 for Colombia, Ecuador, and El Salvador. We compute the national average (solid line) and the minimum-maximum interval (shaded area) across all municipalities for which we have data. Please note that our reported values indicate reductions in movement, so positive quantities should be interpreted as decreases in mobility. For a qualitative comparison we also report the Stringency Index from the Oxford COVID-19 Government Response Tracker [39] which measures the strictness of policies implemented to contain COVID-19 in the three countries (see Sect. 4.1). During March 2020 mobility dropped sharply reaching a maximum reduction in late March/early April (53% for Colombia, 64% for Ecuador and El Salvador). This drop matches the introduction of increasingly tougher measures, as shown by the evolution of the Stringency Index in the three countries. After reaching the maximum, we observe an inversion in the mobility which anticipates, and then follows, the relaxing of NPIs in early summer. The movement reduction approaches the pre-pandemic baseline (i.e., $\sim 0\%$ reduction) by late 2020.

It is important to note how heterogeneities within each country do exist. At the peak of mobility drops in Colombia, for example, the municipality associated with the minimum change shows a mobility reduction of 24%. In the same week, the municipality featuring the strongest decline has a reduction of 73%, marking an absolute minimum-maximum difference of 49%. We note similar, though less pronounced, patterns also for Ecuador and El Salvador (minimum-maximum absolute difference of, respectively, 31% and 25%).

The *Movement Range Maps* dataset provides also a second mobility metric describing the fraction of individuals that appear to stay within a small area for the whole day. The two mobility metrics provide different pieces of information, but they can be considered complementary. The overall change in mobility is generally used in similar research [1]. It provides a more comprehensive and nuanced view about the impact on NPIs on mobility allowing, for example, to appreciate tendencies towards shorter and less frequent trips. Therefore, in the main text, we will carry out analyses only for the *movement change* metric, while we refer the reader to the Additional file 1 for the analogous analyses for the second mobility metric. The overall picture emerging from both metric is coherent.

### Proxies of digital infrastructure quality

We measure the quality of the digital infrastructure using as a proxy internet Speedtest® results provided by Ookla® [40]. Ookla’s Speedtest Intelligence® solution offers analysis of internet performance metrics, such as connection data rate [52]. The tests are geolocalized and provide download/upload speed (expressed in Megabits per second) and latency (in milliseconds) for fixed networks. For the purpose of this analysis, we characterize the quality of digital infrastructure using fixed download speed. Our dataset includes more than 90M Speedtest® measurements among the three countries. For each municipality in the three countries, we compute a static (i.e., fixed in the temporal horizon of our analysis) metric. We refer the reader to Sect. 4.1 for more details.

**Figure 2:**
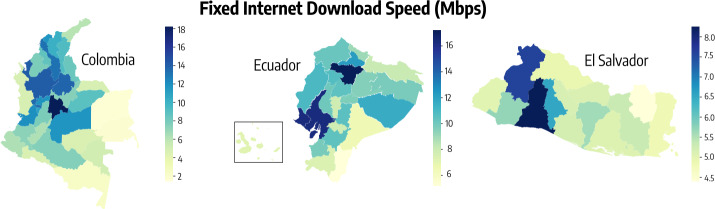
Fixed internet quality in Colombia, Ecuador, and El Salvador (2019/2020). Average fixed internet download speed (expressed in Mbps) computed from Ookla® Speedtest Intelligence® data for different departments of Colombia, Ecuador, and El Salvador

In Fig. 2 we show the average download fixed internet speed (expressed in Megabits per second) in different departments of Colombia, Ecuador, and El Salvador. From the plot we observe that the quality of digital infrastructure varies widely across regions within the same country. In El Salvador, the average download speed in *La Libertad* is nearly double that in *Morazán*. In Colombia, the department with the best infrastructure shows a ∼13 times higher download speed with respect to the one with the worst network. In Ecuador, the ratio between higher and lower speed among departments is ∼3.

Considering the emphasis of such metric in our analysis, it is fundamental to notice some limitations of testing speed as a proxy of the digital infrastructure. Indeed, the outcome of an individual’s test might differ from the real internet speed. A recent review on the subject highlights some of the key issues [52]. Some are rather technical aspects linked to bottlenecks in home networks (i.e., Wi-Fi routers), the details of the software or tool used to make a measurement, and the number of devices sharing the connection. Others factors instead are linked to the users themselves. In fact, their tests might not reflect the average speed since they might be done when a user is experiencing connectivity issues or needs to know the level of connectivity available in a area that is new to them. Furthermore, only users more digitally aware know about these types of services. These factors induce undoubtedly some self-selection biases in the sample. However, Ookla® is considered as a *canonical* network performance testing service widely used to infer the features of internet connectivity across and within regions by academic and official institutions [31, 52, 53]. Also, our analysis aggregates Speedtest® measurements at the level of municipality. In doing so, some of the more technical issues mentioned above are averaged across many different instances. Furthermore, our analyses are not dependent on the absolute value of aggregated connectivity but on the differences across municipalities. Overall, it is important to acknowledge how the data used here is only a proxy of the digital infrastructure quality.

### Association between mobility change and digital infrastructure quality

Considering the novelty of the metric in this context, before moving to a more rigorous analysis, we first investigate the association between changes in mobility and the quality of the digital infrastructure in different municipalities.

**Figure 3:**
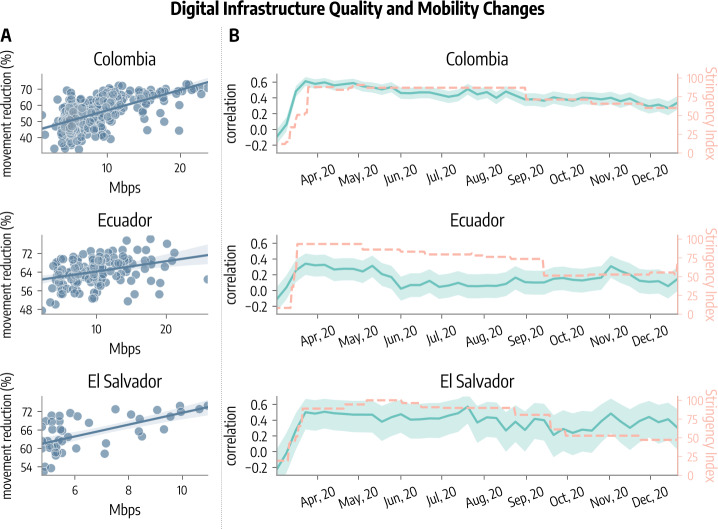
Association between mobility reduction and digital infrastructure quality in different municipalities of Colombia, Ecuador, and El Salvador. (**A**) We plot the greatest reduction in mobility against the average download speed in different municipalities. (**B**) We plot the Pearson correlation coefficient (median and 95% CI) between average weekly movement reduction and average download speed of different municipalities. The orange dashed lines in all plots represent the Stringency Index. Confidence intervals for correlation coefficients are obtained from $p_{values}$ setting a significance level $\alpha =5\%$

In Fig. 3-A we plot the maximum percentage reduction in mobility (i.e., the greatest level of compliance to NPIs) against the average fixed download speed of each municipality in the three countries. The plot reveals a significant positive linear correlation between the two quantities. Indeed, we obtain Pearson’s correlation coefficient of 0.62 (95% CI: $[0.55; 0.68]$) for Colombia, 0.34 (95% CI: $[0.19; 0.47]$) for Ecuador, and 0.61 (95% CI: $[0.39; 0.76]$) for El Salvador. This indicates that 38%, 12%, and 40% of the variance of the greatest drawdown in movements among municipalities in respectively Colombia, Ecuador, and El Salvador is explained by the quality of the digital infrastructure expressed as average fixed download speed. The slopes of the regression lines reported in the figure indicate that as download speed increases by 10 Megabits, the maximum mobility reduction increases by another 13% in Colombia, 4% in Ecuador, and 19% in El Salvador. Hence, this first finding suggests that municipalities with higher digital infrastructure quality are also those where mobility has changed (reduced) more.

In Fig. 3-B we show the temporal evolution of the Pearson correlation coefficient between weekly mobility change and average fixed download speed of different municipalities in Colombia, Ecuador, and El Salvador. As expected from the previous analysis, we obtain positive correlations. More interestingly, we notice that, across the board, correlations tend to follow the Stringency Index, shown in the figure as an orange dashed line. This trend is particularly prominent in Colombia, whereas it is less pronounced in El Salvador and Ecuador, possibly due to wider confidence intervals in the latter countries. In early/mid March 2020, when the first restrictions were imposed, correlations have a significant jump, reaching a peak close to the peak of restrictions. Afterward, correlations tend to decrease together with the Stringency Index as some restrictions are partially lifted. For example, average correlations in April 2020 are 0.58, 0.29, and 0.49 for Colombia, Ecuador, and El Salvador respectively, while they drop to 0.31, 0.11, 0.36 in December 2020, marking a $-47\%$, $-62\%$, $-25\%$ decline.

When interpreting this result is important to notice several facts. The evolution of the correlation after the peak follows a slow decreasing trend with respect to the reduction in mobility observed in Fig. 1. However, the correlation is not dependent on the scale of the variables. Even when the overall mobility reduction is small, the ordering (effect) of (on) municipalities according to this metric may still be strongly related to internet speed. Correlation does not imply causation. To highlight this aspect we have opted for the use of the word “association” between the two quantities. Furthermore, there may be an association between these two quantities because both are correlated to other confounders. For example, previous works have shown that the adherence to NPIs of different communities correlates with their socio-economic status [11–14]. At the same time, better network coverage might be correlated with higher socio-economic conditions. Indeed, a reliable internet access and investments in infrastructure favor human and economic growth [54–56]. As a result, movement changes and network quality may show significant correlations because both are directly influenced by the socio-economic status of the municipality. We tackle the issue of confounders in three ways. First, we make use of partial correlations. We compute the Pearson partial correlation between movement reduction and network quality controlling for the socio-economic status of different municipalities [57]. We consider the Relative Wealth Index publicly available as part of Meta’s Data for Good program as a proxy for the socio-economic status of different municipalities (see Sect. 4.1 for details). The correlations presented in Fig. 3-A, after controlling for the socio-economic status, become 0.32 (95% CI: $[0.23, 0.42]$), 0.29 (95% CI: $[0.14, 0.43]$), 0.40 (95% CI: $[0.13, 0.62]$) for Colombia, Ecuador, and El Salvador respectively. We notice how the partial correlation is smaller but still significant and showing positive sign. We obtain wider confidence intervals in the case of El Salvador due to the smaller sample size. Indeed, we have data for 459 municipalities in Colombia, 164 in Ecuador, and 56 in El Salvador. In the Additional file 1, we report more details about the methodology of partial correlations and we repeat the analysis using also other features as controls. We find that the overall picture remains unaltered.

Second, we conduct a mediation analysis [58]. In particular, we study the potential role of the Relative Wealth Index as mediator of the association between reduction of mobility and quality of the internet infrastructure. The results, detailed in the Additional file 1, confirm that, across the three countries, the Relative Wealth Index acts only as partial mediator, thus accounting only for some of the association between the reduction in mobility and the digital infrastructure quality. Hence, the latter variable has still a direct effect on the compliance with NPIs.

As a third and more systematic step, in the next section, we investigate the link between the two quantities by using regression techniques with many additional features to identify the effects and importance of each factor.

### Regression analysis

We perform regression analyses to study the extent to which different municipalities changed mobility as a function of several independent variables. We first use a static approach in which the dependent variable is the maximum movement reduction observed in 2020 for different municipalities (see Fig. 3-A). Besides the average download speed (*Mbps*) that we introduced previously and that acts as a proxy of the digital infrastructure quality, we consider several additional independent variables that we can classify in three categories:

- socio-demographic features: total population (*population*), population density (*density*), fraction of over 60 (60+);
- socio-economic features: GDP per capita (*GDP*), relative wealth index (*RWI*);
- epidemiological indicators: total number of cases per 100k reported in the week of the maximum movement reduction (*cases*).

**Figure 4:**
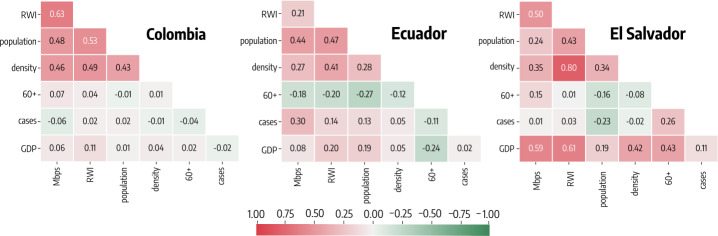
Correlations between independent features

Before moving forward, we investigate the correlation between independent variables. In Fig. 4 we show the Pearson correlation between covariates for the three countries. We obtain a maximum absolute coefficient of 0.63 between *RWI* and *Mbps* in Colombia, of 0.48 between *population* and *RWI* in Ecuador, and of 0.80 between *density* and *RWI* in El Salvador. As expected, some of the variables are indeed correlated. In order to access how such correlations might affect the regression we use the maximum variance inflation factor which quantifies the multicollinearity between variables [59]. We obtain 2.01, 1.59, and 4.31 for Colombia, Ecuador and El Salvador which indicate moderately low multicollinearity. Values above 5 are customarily used to flag multicollinearity problems.

In the main text, we use ordinary least squares, but in the Additional file 1 we repeat analyses using regularized regressions (i.e., ridge regression) and bootstrapping. Regularized regressions in particular are better suited to limit the issues of multicollinearity, which may cause inflated standard errors and decreased statistical power of the coefficients. The results across all methods used are consistent. All features (independent and dependent) are standardized to their distribution (i.e., they are transformed to have a mean of 0 and a standard deviation of 1). Full details on data, pre-processing, model and estimation are provided in the Materials and Methods section and the Additional file 1.

**Figure 5:**
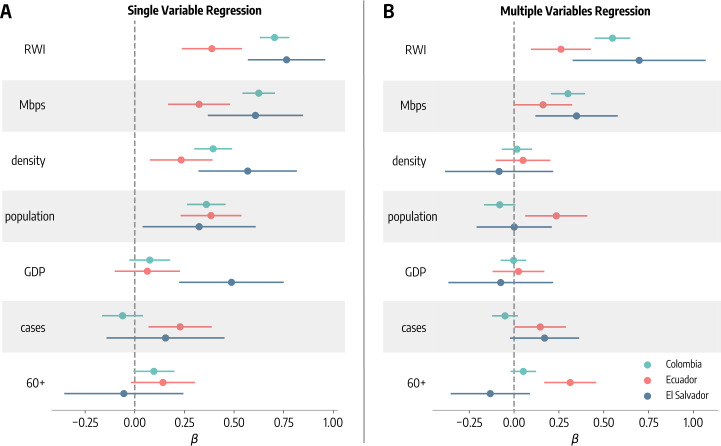
Coefficients of the regressions (maximum movement reduction as dependent variable). (**A**) Coefficients of the single variable regressions (median and 95% confidence intervals). (**B**) Coefficients of the multiple variables regression (median and 95% confidence intervals). The regression model considers standardized regressors, therefore the coefficients are also standardized and represent the change in the response variable associated with a one standard deviation change in the corresponding predictor variable

We use both single and multiple variables regressions. In the single variable analysis, we simply regress each feature singularly against the maximum movement reduction of different municipalities. Estimated standardized coefficients (medians and 95% confidence intervals) are reported in Fig. 5-A. The relative wealth index has a significant positive coefficient in all three countries. The GDP per capita coefficient is also positive but is significant only for El Salvador. This finding confirms the critical role of socio-economic attributes in NPIs adherence reported in previous works [11, 13, 60–62]. Looking at socio-demographic features, we find that both population and density have a positive significant coefficient in all countries. This is in line with previous findings that have shown how individuals living in larger and denser areas had more pronounced mobility reductions [60, 63]. An older population has been observed to be associated with higher mobility reductions [60, 63]. However, in our results the role of the fraction of 60+ people is less clear, showing a positive but barely significant coefficient in the case of Colombia and Ecuador. The number of reported cases per 100k has a positive and significant effect only for Ecuador. This is line with previous research where higher attack rates were found to be associated with stronger mobility reductions [63]. The small influence of reported cases may be because the highest mobility drop was reached in different municipalities during March/April 2020 following the introduction of restrictions and grim news coming from other countries rather than local COVID-19 incidence. As a result, most of the municipalities reported very few or no cases in the week when mobility reduction reached its acme. Finally, the single variable regression analysis confirms the significant positive role of average download speed previously underlined through the correlation analysis.

In the multiple variables analysis, the maximum movement reduction is regressed against all features previously introduced (coefficients are reported in Fig. 5-B). The strong importance of the socio-economic status is confirmed by the positive significant coefficient of the relative wealth index in all three countries. Across the board, the *RWI* shows the highest standardized coefficient, therefore it represents the most important feature among those considered. Interestingly, the coefficient of the average download speed remains positive and significant in all three countries. Overall, we note how the coefficient for Ecuador is smaller with respect to the other two countries hinting to a weaker effect of the digital infrastructure quality on NPI adoption. These results, combined with the partial correlations, confirms the importance of digital infrastructure in NPIs adherence even after controlling for several other factors. Other attributes have in general smaller importance when considered in concert with the others. The number of cases has a marginally significant and positive effect only for Ecuador and El Salvador. Population size and fraction of 60+ remain significant only in the case of Ecuador. Overall the regression model has a coefficient of determination ($R^{2}$) of 0.56, 0.34, and 0.69 in respectively Colombia, Ecuador, and El Salvador.

Summarizing the results from the multivariate regression we find that the quality of the digital infrastructure is a significant factor influencing the adoption of NPIs, but it is not the only important variable. In line with previous research, socio-economic features of the population confirm their key role in explaining adherence to NPIs.

As an additional assessment on the role of average download speed, we build a second model by repeating the multiple variables regression without this variable. We compare such restricted model to the full one by using the F-test. The null hypothesis is that the restricted model fits the data better than the model including the quality of the digital infrastructure. For all three countries, we obtain p-values that are smaller than the significance level of 5% ($p_{value}<10^{-3}$ for Colombia, $p_{value}=0.045$ for Ecuador, and $p_{value}=0.003$ for El Salvador), offering evidence against the null hypothesis and showing that the inclusion of internet quality among the regressors improves the model. Additionally, we compute the Akaike Information Criterion (AIC) of the two models with and without internet quality as $AIC = 2 k - 2 log(L)$, where *k* is the number of independent variables and *L* is the likelihood function [64]. Therefore, the $AIC$ considers both model’s complexity and the accordance between model’s estimates and observations. The difference of the AICs of the model with and without download speed as independent variable ($\Delta _{AIC}$) is −38.4, −2.23, −8.5 for Colombia, Ecuador, and El Salvador. As a rule of thumb, a decrease in the AIC of at least 2 units indicates that the model with lower $AIC$ is significantly better [65]. We turn the $\Delta _{AIC}$ into Akaike Weights as $exp(\Delta _{AIC}/2)$ [66]. As shown in Ref. [66], the Akaike Weight $w_{i}$ can be interpreted as the probability that - given the data and the set of candidate models - the associated model $M_{i}$ is the best in the AIC sense (i.e., it minimizes the Kullback–Leibler discrepancy). We obtain that the model without download speed is, for the three countries, <10^−3^, 0.32, and 0.01 times as probable as the model with download speed. Finally, we also estimate that the inclusion of internet speed leads to 9.6%, 5.8%, and 12.0% uplift in $R^{2}$ for respectively Colombia, Ecuador, and El Salvador. These additional findings confirm the influential role of the quality of digital infrastructure in NPIs compliance.

**Figure 6:**
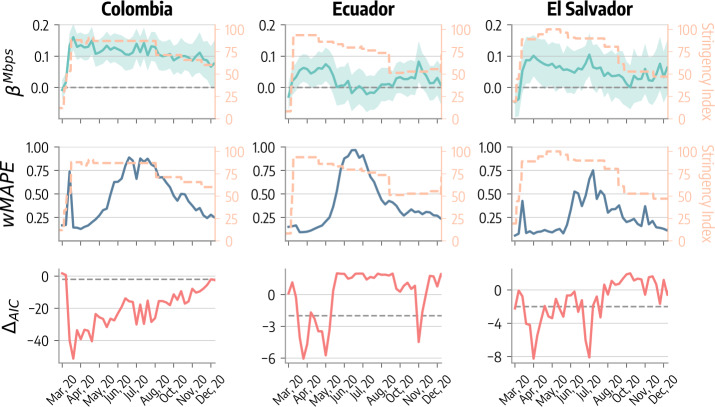
Weekly regression results. First line shows the evolution of the regression coefficient of the average download speed as a function of time for the three countries. Second line shows the weighted mean absolute percentage error *wMAPE* in different weeks. Third line shows the evolution of the $\Delta _{AIC}$ between the model with and without download speed as an independent variable. We average all quantities over a month to rule out the influence of noise. The orange dashed line in the different figures represents the evolution of the Stringency Index

In addition to the static approach, we propose a second regression that explores possible time-varying relationships between dependent and independent variables. For each week of 2020, we repeat the multiple variables regression using however as dependent variable the average reduction in mobility observed in that week across the different municipalities. Also, the independent variable *cases* in this cases refers to the number of weekly cases reported per 100*k* in that week. In Fig. 6 we show the evolution in time of the estimated coefficient for the average download speed ($\beta ^{Mbps}$). We observe a similar trend across the three countries. Indeed, $\beta ^{Mbps}$ is ∼0 at the beginning of March, 2020, then it grows and reaches a maximum around late March/April, concurrently with the peak of restrictive measures (Stringency Index reported in figure as an orange dashed line). In the second row of Fig. 6, we find again a common pattern across the three countries: the weighted mean absolute percentage error (*wMAPE*) tends to be lower when the NPIs in place are stricter (i.e., higher values of the Stringency Index). This indicates that the model is better at predicting mobility changes when measures implemented are stricter. Finally, the third row of the figure reports the time evolution of the $\Delta _{AIC}$ between the model with and without *Mbps* as independent variable. The figure confirms the importance of the variable, especially during the early months of 2020, where we obtain $\Delta _{AIC}$ values that are smaller than −2.

## Conclusions

The burden of the COVID-19 Pandemic and of the measures put in place to fight it are distributed unequally across different social, economic, and demographic backgrounds [1, 11–20]. In this work, we studied the relationship between NPIs compliance and its determinants during 2020 in the municipalities of Colombia, Ecuador, and El Salvador. Besides previously studied factors - such as socio-economic status and epidemiological indicators - we explored the role of a barrier affecting NPIs adherence: the quality of the digital infrastructure. In doing so, we tested the hypothesis that improved internet connectivity can reduce the need for travel by allowing individuals to carry out various activities from home. Analyzing a unique dataset including tens of millions of geolocalized internet Speedtests® we found support for the hypothesis showing that municipalities with a better connection were associated with higher mobility reductions. We found that this correlation remains significant also after controlling for confounders, including the socio-economic level of the municipality. By using several regression models we explored the role of other independent variables. We found that mobility reductions were more pronounced in larger, denser, and wealthier municipalities. In addition, these analyses confirmed that the digital infrastructure quality explains part of the differences in NPIs compliance and that its effects are higher at the peak of the strictness of non-pharmaceutical interventions.

These findings corroborate and expand the literature. Previous studies explored - mainly via surveys - the role of internet access and quality in the engagement in specific activities such as telemedicine and homeschooling during the Pandemic. They showed, for example, that poor Internet connectivity at home explains gaps in student performances [38] and that lack of access to digital tools poses a barrier to the adoption of digital health innovations and contributes to poor health outcomes [67]. Interestingly, these works found that these effects remain significant also after controlling for socio-economic attributes. A report focused on the early months of the Pandemic in the US, showed results that are compatible with our findings [32]. The authors used a binary variable to describe the access to high-speed internet and showed that this is a relevant factor for compliance to NPIs following state-level mandates.

The present work comes with limitations. Heterogeneity in the usage of mobile devices can lead to biases in aggregated mobility data such as the Movement Range Maps [68–71]. As shown in the Additional file 1, the average Relative Wealth Index of municipalities for which we have mobility data is generally higher respect to those without mobility data. Hence, our results, as all similar studies based on passive digital data collections, are limited and biased by incomplete and heterogeneous penetration of the technology used. The socio-demographic and socio-economic features we used are only aggregated and do not have access to individual information. Our characterization of compliance with NPIs is based solely on mobility data, thus overlooking adherence to other measures that are not strictly related to mobility, such as face masks adoption. However, our analysis focused on the period of March-December 2020, during which restrictive measures that impacted human mobility were a critical component of NPIs. Furthermore, this period was unfortunately characterized by uncertainties about the routes of infection and by a global scarce availability of personal protective equipment (PPE) such as face masks which resulted in limited adoption of such measures. Additionally, we hypothesize that access to a better internet connectivity could reduce the need for travel by enabling remote activities, thereby affecting adherence to NPIs targeting mobility to a greater extent. For these reasons, while recognizing the potential limitations, we believe that mobility data provides a good proxy for NPIs adherence in the period under study. As explained before, also Ookla® data presents limitations. Indeed, the result of an individual’s test might differ from the real internet speed [52]. However, here we do not aim to provide an accurate representation of the quality of the digital infrastructure but rather characterize the differences between municipalities. Therefore, thanks to the relative nature of the analyses, key issues of tests do not represent a concern. Another possible bias may be introduced by the heterogeneous testing behavior of individuals. Indeed, some may perform a Speedtest® much more frequently than others. We solve this by averaging results from a single user before further aggregation, as explained in the Materials and Methods section. We used only data proxy of fixed Internet lines disregarding mobile connections. The number of mobile tests is a small fraction of fixed tests (around 6%). Furthermore, the average mobile internet connections we observe in the data are too low to support video calls and other tools that would allow carrying on many activities from home reducing travels. We leave exploring and contrasting similarities as well as differences between fixed and mobile connections to future work. While we controlled for several factors known to play a role in NPIs adherence, part of the results may be also explained by omitted variables. On top of these, we mention information about formal employment and labor structure [60] that, because of lack of data, we did not model explicitly. In the Additional file 1, we explore this dimension by repeating analyses with additional features for Colombia. We find that even after including labor formality and relative importance of different economic sectors among the independent variables of the regressions, digital infrastructure quality is still a significant factor explaining NPIs adherence. We only considered the quality of digital infrastructure. Arguably, also the adoption of digital tools is an important factor playing a role in NPIs adherence. However, the adoption of digital tools is affected by a wider range of factors such as many behavioral tendencies and aspects of digital literacy that are hard to assess or disentangle. Furthermore, there is a scarce availability of updated data on digital tools adoption (especially for El Salvador and Ecuador) at the spatial level of our analysis (i.e., municipalities). Hence in this work, we decided to focus mainly on the quality of the digital infrastructure and its possible impact on NPIs adherence. Nonetheless, the two aspects are not unrelated. As shown in the Additional file 1, in the case of Colombian municipalities, we find a strong positive correlation between infrastructure quality and internet penetration. Furthermore, across the three countries, internet speed and number of tests are positively correlated. In the Additional file 1, we test the role of digital tools adoption in NPIs compliance proposing as proxies the number of Speedtest® measurements performed (for all the three countries) and the number of internet subscriptions (only for Colombia) in different municipalities. The results indicate the variables capturing adoption of digital tools as important and significant. Furthermore, we repeated the regression considering the number of tests (proxy of digital adoption) instead of the average speed (proxy of digital infrastructure quality) finding comparable results for the three countries. Overall, these results show how, together with digital infrastructure quality, also the adoption of digital tools is a significant determinant of NPIs adherence. As epidemic indicator, possibly affecting adoption of NPIs, we adopted the number of cases. Especially in the early phases of the pandemic, the virus spread cryptically (i.e., largely undetected) [72]. Hence, the cases reported by surveillance systems offer only a partial picture. The number of deaths is often a preferred metric. However, in the countries under study here, the initial set of strict NPIs has been implemented as result of the news coming from other countries in Asia, Europe and the US rather than due to a rapid growth of local deaths. Hence, at the height of the initial set of NPIs in March/April 2020, many municipalites recorded zero deaths. Finally, we are aware of spatial heterogeneities, at the levels of municipalities and regions, in NPIs that might have affected communities within each country differently [60]. However, we do not have detailed data reconstructing the timeline of NPIs at this resolution. For this reason, while we expect the stringency of NPIs to affect the mobility within each municipality, we did not include such variables in the regression. We leave this extension to future work.

In conclusion, our work confirms the role of socio-economic and socio-demographic factors in NPIs compliance and sheds light on possible barriers to adoption represented by the heterogeneous digital infrastructure quality and the digital divide. Our study also demonstrates the potential use of non-traditional data sources, such as internet Speedtests®, to investigate the adherence to NPIs in countries that have limited information available due to resource constraints. Our results call for policies and targeted investments aimed at closing the digital gap, improving network reliability as well as equality across communities.

## Materials and methods

### Datasets

*Oxford COVID-19 Government Response Tracker.* We consider the Stringency Index from the Oxford COVID-19 Government Response Tracker to quantify the strictness of policies implemented to curb SARS-CoV-2 spread [39]. This dataset compiles information from various data sources to generate standardized policy indicators that track which governments have taken which measures, and when. The Stringency Index goes from a minimum of 0 (i.e., no measure) to a maximum of 100 (i.e., strictest measures). It is computed as the average of several sub-indicators that describe, for example, the level of closure of schools and workplaces, restrictions to international travel, stay at home requirements, and the implementation of public health information campaign. For more details on the methodology refer to Ref. [73]. The Stringency Index has daily resolution and is available at the country level for Colombia, Ecuador, and El Salvador. In the Additional file 1 we include a visualization of some of the sub-indices that contribute to the Stringency index.

*Movement Range Maps.* To estimate adherence to NPIs we consider changes in the mobility captured in the dataset *Movement Range Maps* from Meta’s Data for Good Program [46]. This dataset is publicly available and has been utilized in the past to analyze changes in mobility patterns, as well as to evaluate how various communities responded to physical distancing measures implemented to combat the COVID-19 pandemic. [15, 74, 75]. It provides two main metrics: i) a percentage reduction in movement computed with respect to a pre-pandemic baseline, and ii) the fraction of individuals that appear to stay within a small area for the whole day. The two metrics are computed using de-identified data of Facebook users who opt-in for Location History and background location collection. To safeguard the privacy of its users, Meta excludes areas with low population density and adds an appropriate level of noise before sharing the data. This ensures that re-identification of individuals is not possible. We refer the reader to Ref. [76] for more details on the methodology used by Meta to generate the *Movement Range Maps*. The data are available for several countries at the municipal level (GADM2) and have a temporal resolution of the day. We have data for 459 of the 1065 GADM2 areas (i.e., municipalities) in Colombia, for 164 of the 223 in Ecuador, and for 56 of the 266 in El Salvador. The spatial distribution of municipalities for which data are available is shown in the Additional file 1. We have data for at least one municipality in each of the 32, 24, and 14 GADM1 areas (i.e., regions) in, respectively, Colombia, Ecuador, and El Salvador. In the analyses presented above, we performed different aggregations of this data. First, we have excluded weekends from these calculations due to their anomalous behavior, which can be attributed to the fact that the baseline used for comparison is not specific to weekends. We compute the movement reduction in municipality *m* during week $t_{w}$ as:

$$ MR^{m}(t_{w}) = \frac{1}{n_{w}}\sum _{i=1}^{n_{w}} MR^{m}(t_{i}) $$

Where $t_{i}$ are the days in week $t_{w}$ and $n_{w}$ is the number of days in week $t_{w}$. Here we consider only municipalities for which the whole week Monday to Friday was available, therefore $n_{w}=5$. The maximum movement reduction for each municipality, that we used both in the correlation and the regression analysis, is simply computed as:

$$ MR^{m}_{max} = \max_{t_{w}} \bigl\{ MR^{m}(t_{w})\bigr\} $$

Finally, national weekly movement reductions shown in Fig. 1 are computed averaging the weekly movement reduction of single municipalities:

$$ MR(t_{w})= \frac{1}{N}\sum_{m=1}^{M} MR^{m}(t_{w}) $$

Where *M* is the number of municipalities in the country considered.

*Relative Wealth Index.* We characterize the socio-economic status of different GADM2 areas using the Relative Wealth Index (RWI) from Meta’s Data for Good Program. The index, publicly released in 2021, provides micro-estimates of the relative standard of living within countries. The index is constructed by utilizing nontraditional data sources such as satellite imagery and privacy-preserving Facebook connectivity data. To validate the accuracy of the index, Meta uses ground truth measurements obtained from the Demographic and Health Surveys. We refer the reader to Ref. [77] for more details on the index calculation. The RWI is available for nearly 93 low and middle income worldwide at a very high spatial resolution (30*m* population density tiles). Here, we follow the procedure described in Ref. [78] to aggregate the RWI at the municipal level. First, we compute which administrative area (i.e., municipality) contains the centroid of each RWI 30*m* tile. Upon completion of this step, each municipality will be linked with a corresponding set of tiles, each of which will have an RWI value associated with it. The *High-Resolution Population Density Maps and Demographic Estimates* from Meta’s Data for Good program [79] provides a population estimate for each of this tile. Therefore, we compute the RWI of municipality *m* taking the weighted average according to the population of tiles within *m*:

$$ RWI^{m} = \frac{1}{P_{m}}\sum_{i \in m} P_{i} RWI_{i} $$

Where $P_{m}$ is the total population of *m* and $P_{i}$ is the population of each tile *i* contained in *m*. In the Additional file 1, we compare the RWI against more traditional measures of wealth for the countries considered, namely the Multidimensional Poverty Index for Colombia and the Human Development Index for Ecuador and El Salvador. We find high and significant correlations between the RWI and these measures in all three cases.

*Speedtest® data.* We characterize the quality of digital infrastructure using as proxy Speedtest Intelligence data® by Ookla® [40]. Speedtest® apps offer free analyses of Internet access performance metrics, such as connection data rate. The tests are geolocalized and provide download/upload speed (expressed in Megabits per second), and latency (in milliseconds) for fixed network. In this study, we focused on download speed as a metric to assess the quality of digital infrastructure, since it is the commonly used measure among the three. The dataset includes $65{,}863{,}831$ tests for Colombia, $4{,}821{,}878$ for El Salvador, and $22{,}159{,}403$ for Ecuador performed during 2019-2020. Before computing any statistics we clean the data as follows. First, we exclude all tests showing 0 *Mbps* download speed since these are generally tests that failed and are not informative of the real network quality. Second, to remove the influence of outliers we exclude tests that show a download speed higher than 2 Gigabits per second, as this is considered as the maximum value for broadband technology. Therefore, values exceeding this threshold are deemed anomalous and may be associated with inaccuracies in the measurement process. Lastly, we exclude tests that show a latency higher than the 95th percentile, since these values are considered outliers and not informative on the actual quality of the digital infrastructure. The cleaning process leads to the exclusion of around 5% of the total tests. After preprocessing, we compute the average download speed for a given municipality *m* as follows. First we select all speedtests that were performed in *m*. Second, we take the median of the results of tests performed by a single user. That is, to each user *u* we will have an associated download speed computed as:

$$ Mbps^{u, m} = med_{i}\bigl(Mbps_{i}^{u, m} \bigr) $$

This is done to avoid over-representation of users using the service more often than others. Finally, the download speed associated to municipality *m* is simply computed as the median download speed across all users:

$$ Mbps^{m} = med_{u}\bigl(Mbps^{u, m}\bigr) $$

*Population data.* For Colombia we get the population of different municipalities from the official census [80]. Because of the lack of updated data on municipality population in Ecuador and El Salvador, we consider the publicly available *High-Resolution Population Density Maps and Demographic Estimates* from Meta’s Data for Good program [79]. This dataset provides accurate population data using satellite imagery and census data. Besides total population count, it also provides demographic breakdown, including the number of people over 60, which we use to compute the fraction of 60+ for all three countries used in the regression analysis of Sect. 2.4. The population density is calculated as the ratio of the total population count and the estimated area of the municipality. The estimated area is obtained by computing the area of the polygon associated with the municipality in the shape file used for geographic data manipulation (refer to the sources below for geographic data).

*COVID-19 cases.* We consider official epidemiological sources for Colombia [81], Ecuador [82], and El Salvador [83]. The data provides the number of notified COVID-19 cases for each municipality on a daily basis.

*GDP data.* We get GDP data from Ref. [84] for Colombia, from Ref. [85] for Ecuador, and from Ref. [86] for El Salvador. For comparison across countries, all GDP figures are expressed in US dollars.

*Geographic data.* We download spatial data for the three countries and their sub-divisions from the Database of Global Administrative Areas [87].

## Supplementary Information

Below is the link to the electronic supplementary material.

Supplementary information (PDF 8.7 MB)

## Abbreviations

Mbps: Megabits per second
population: total population
density: population density
60+: fraction of over 60
GDP: Gross Domestic Product per capita
RWI: relative wealth index
cases: total number of cases per 100k reported in the week of the maximum movement reduction
