# Supplementary material for: The adoption of non-pharmaceutical interventions and the role of digital infrastructure during the COVID-19 pandemic in Colombia, Ecuador, and El Salvador
Source: EPJ Data Sci. 2023 Jun 6;12(1):18. doi: 10.1140/epjds/s13688-023-00395-5 (PMC10243255; doi:10.1140/epjds/s13688-023-00395-5)
Supplement: Supplementary file 1 — Supplementary information (PDF 8.7 MB) [file 13688_2023_395_MOESM1_ESM.pdf]

# Supplementary Information for *The adoption of non-pharmaceutical interventions and the role of digital infrastructure during the COVID-19 Pandemic in Colombia, Ecuador, and El Salvador*

Nicolò Gozzi[1,2], Niccolò Comini[3], Nicola Perra[4]

[1] Networks and Urban Systems Centre, University of Greenwich, UK
[2] ISI Foundation, Turin, Italy
[3] The World Bank
[4] School of Mathematical Sciences, Queen Mary University of London, UK

April 13, 2023

## Contents

**1 Relative Wealth Index** **2**

**2 Geographic coverage of Movement Range Maps** **3**

**3 Timeline of NPIs implemented in Colombia, Ecuador, and El Salvador** **4**

**4 Partial correlations** **5**

**5 Mediation analysis** **6**

**6 Regularized linear models and bootstrapping** **7**
6.1 Ridge regression . . . 7
6.2 Bootstrap sampling . . . 7
6.3 Multiple variable regression with Ridge and bootstrap sampling . . . 7

**7 The *Stay at Home* mobility metric** **9**
7.1 The metric . . . 9
7.2 Correlations . . . 9
7.3 Regressions . . . 9

**8 Digital tools adoption and NPIs adherence** **12**
8.1 Internet adoption and infrastructure quality . . . 12
8.2 Proxies of digital tools adoption . . . 12
8.3 Correlations between digital tools adoption and NPIs adherence . . . 12
8.4 Regression analysis . . . 13

**9 Formal employment and labor structure** **16**

# 1 Relative Wealth Index

In Fig. 1 we provide a comparison of the Relative Wealth Index ($RWI$) against more traditional measures of wealth. More in detail, we compare it to the multidimensional poverty index ($MPI$) for Colombia (2018) [1] and to the Human Development Index ($HDI$) of municipalities in Ecuador (2016) [2] and El Salvador (2011) [3]. The MPI is a poverty index developed by United Nations Development Programme (UNDP) in collaboration with the Oxford Poverty and Human Development Initiative (OPHI) that considers multiple dimensions of poverty, such as health, education, and living standards, going beyond income-based measures [4]. It ranges from 0 to 100, with higher values indicating more significant levels of poverty. The HDI is a composite index developed by the UNDP that measures and compares levels of human development among different geographies [5]. It considers factors such as life expectancy, education, and income per capita. It ranges from 0 to 1, with higher values indicating greater levels of human development. Although these measures have different definitions and express different pieces of information, in both cases we find a high significant Pearson correlation coefficient between them and the RWI of different municipalities: $\rho = -0.59$ ($[-0.63, -0.55]$) in the case of Colombia, $\rho = 0.58$ ($[0.49, 0.66]$) in Ecuador, and $\rho = 0.77$ ($[0.71, 0.81]$) in El Salvador. The negative sign obtained for Colombia is expected, given the MPI definition described above.

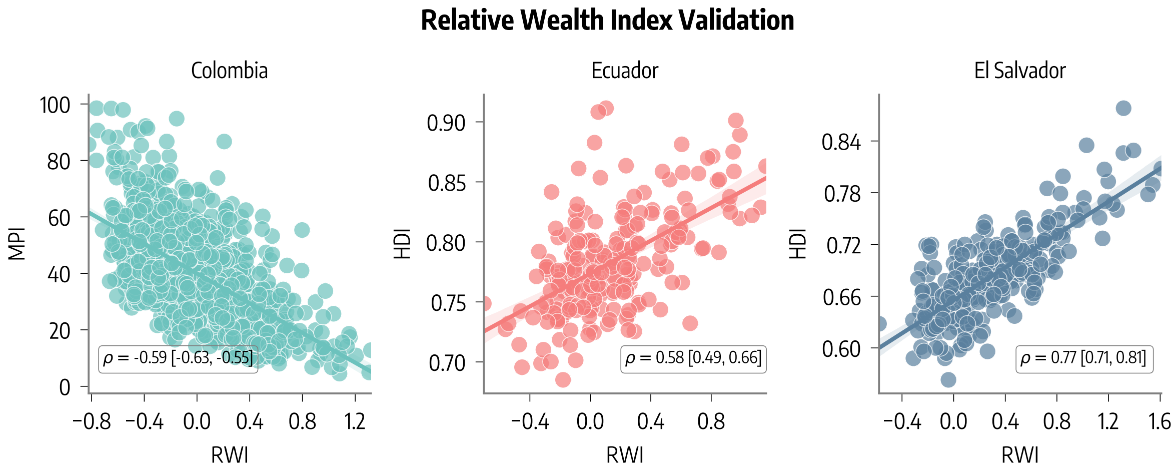


Figure 1: **Validation of Relative Wealth Index.** We show the correlation of the relative wealth index with the multidimensional poverty index (Colombia) and the human development index (Ecuador and El Salvador).

## 2 Geographic coverage of Movement Range Maps

In Fig. 2 we show the geographic coverage of the Meta's Movement Range Maps in the three countries of focus, Colombia, Ecuador, and El Salvador. As discussed in the main text, the metrics featured in this dataset are derived from de-identified data of Facebook users, specifically those who opt-in for Location History and background location collection. To ensure that individuals cannot be re-identified from the data, Meta excludes areas with low population density. Municipalities for which data are not available are colored in grey. We have data for 459 of the 1065 GADM2 areas (i.e., municipalities) in Colombia, for 164 of the 223 in Ecuador, and for 56 of the 266 in El Salvador. More in detail, at least one municipality in each of the 32, 24, and 14 GADM1 areas (i.e., regions) in the three countries is included in the dataset. We note that the average Relative Wealth Index of municipalities for which we have mobility data is higher than that of those without mobility data (0.10 vs −0.24 for Colombia, 0.14 vs −0.11 for Ecuador, and 0.59 vs 0.13 for El Salvador). Therefore, our analyses, similarly to other studies based on passively collected digital data, are limited by heterogeneous technology adoption.

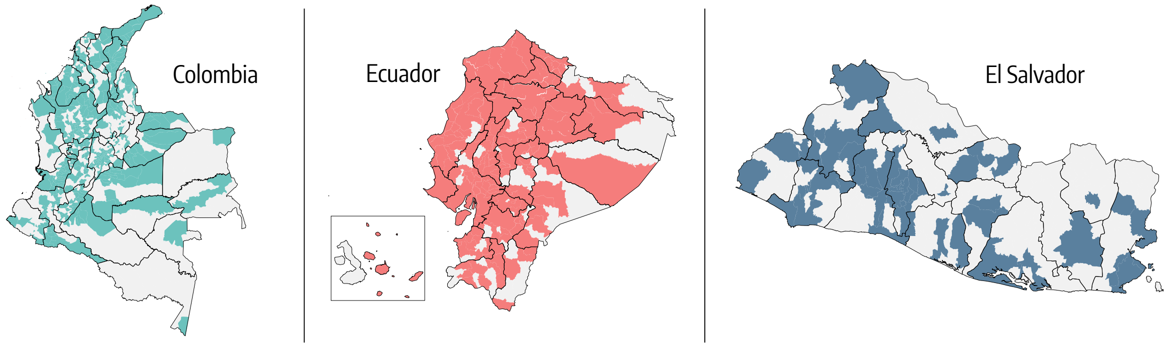


Figure 2: **Geographic coverage of Range Maps in Colombia, Ecuador, and El Salvador.**

# 3 Timeline of NPIs implemented in Colombia, Ecuador, and El Salvador

In the main text, we utilized the Stringency Index from the Oxford COVID-19 Government Response Tracker [6] to assess the strictness of countermeasures implemented by the three countries of interest to contain the spread of COVID-19. In Fig. 3, we present the temporal evolution of four sub-indicators that contribute to the overall Stringency Index, including internal movement restrictions, stay-at-home requirements, and the closure of workplaces and schools. The plot reveals that, in mid-March 2020, all three countries swiftly implemented NPIs aimed at limiting internal movements. At the same time, they also enforced some form of stay-at-home requirements, although this policy was less strict in Colombia and Ecuador. In these countries, people were permitted to leave their homes for activities such as grocery shopping, daily exercise, and essential trips, even after mid-March 2020. In El Salvador, individuals were allowed to leave their homes with minimal exceptions until mid-June 2020, when this measure was relaxed to a level similar to that of the other two countries. Colombia advised the closure of workplaces towards the end of February, and subsequently, in late March, it mandated the closure of all non-essential workplaces, adopting the same measure as Ecuador and El Salvador. Furthermore, all three countries enforced the closure of schools from mid-March onwards.

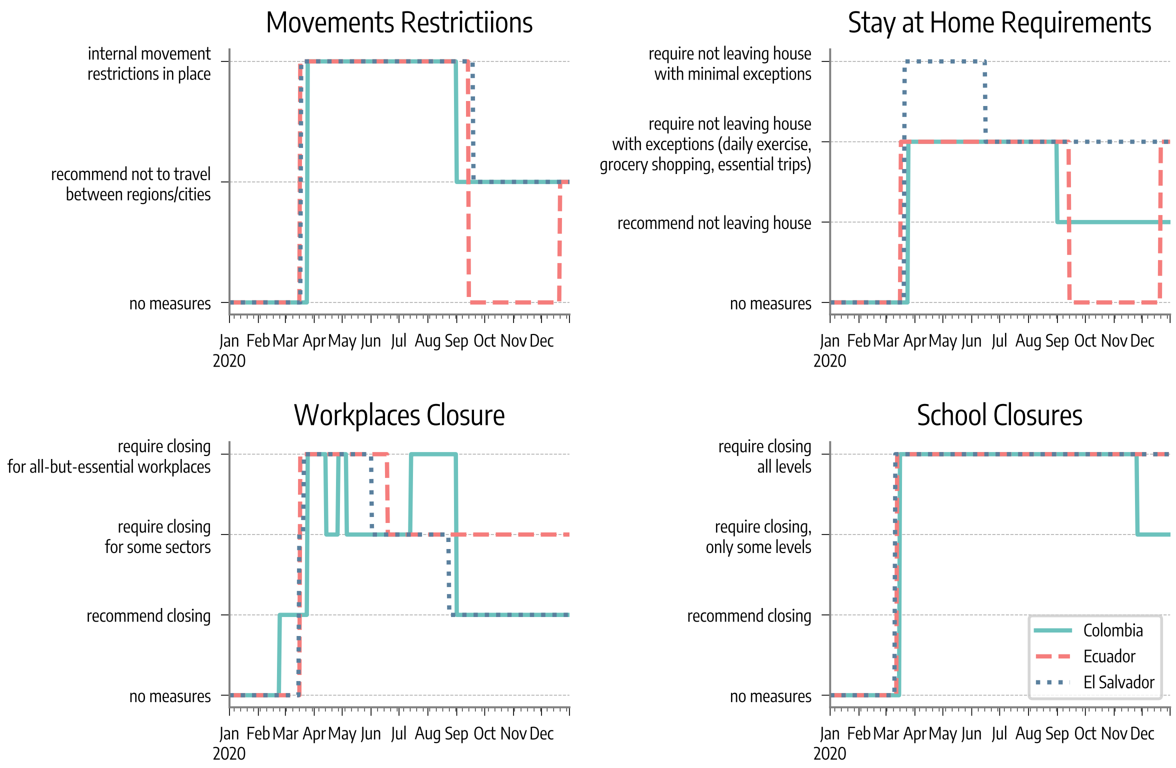


Figure 3: **Timeline of NPIs implemented in Colombia, Ecuador, and El Salvador**. We show the sub-indices on movement restrictions, stay at home requirements, workplaces and school closures provided by the Oxford COVID-19 Government Response Tracker.

# 4 Partial correlations

Consider three random variables $X$, $Y$, and $Z$. Suppose that both $X$ and $Y$ are correlated to $Z$ and that we are interested in finding the association between $X$ and $Y$ accounting for the fact that are both correlated to $Z$. Partial correlation answers to this problem: indeed, it can be regarded as the association between two variables ($X$ and $Y$) after removing the effect of a third variable ($Z$) [7]. In practice, partial correlation is obtained by computing the correlation between the residuals of the two regressions $X$ on $Z$ and $Y$ on $Z$. Like other correlation coefficients, the partial correlation assumes values between $-1$ (perfect negative association) and $+1$ (perfect positive association). In this work, we used the Python library *pingouin* to compute partial correlations [8].

In the main text, we used partial correlations to find the association between the maximum movement reductions and the average download speed of different municipalities controlling for their socioeconomic status using the Relative Wealth Index. Here we repeat this analysis using several other features as control, namely the GDP per capita, the fraction of 60+, the population size, and the population density. Results are reported in Tab. 1. The first row shows the simple Pearson correlation coefficient without any control. Each of the following rows reports the partial correlation using different attributes as control. The last row shows the partial correlation using all the previously listed features as control. We see that, also after controlling for several other features, the residual correlation between movement reduction and digital infrastructure quality remains positive and significant in all cases.

| | **Colombia** | **Ecuador** | **El Salvador** |
|---:|:---:|:---:|:---:|
| / | 0.62 [0.55; 0.68] | 0.34 [0.19; 0.47] | 0.61 [0.39; 0.76] |
| *RWI* | 0.32 [0.23; 0.41] | 0.29 [0.14; 0.43] | 0.4 [0.13; 0.62] |
| *GDP* | 0.62 [0.55; 0.68] | 0.32 [0.17; 0.46] | 0.45 [0.19; 0.66] |
| *60+* | 0.59 [0.52; 0.65] | 0.27 [0.12; 0.41] | 0.57 [0.34; 0.74] |
| *density* | 0.54 [0.46; 0.61] | 0.29 [0.14; 0.43] | 0.53 [0.29; 0.71] |
| *population* | 0.59 [0.52; 0.65] | 0.28 [0.12; 0.42] | 0.58 [0.35; 0.74] |
| *all* | 0.31 [0.22; 0.4] | 0.25 [0.09; 0.39] | 0.45 [0.18; 0.66] |

Table 1: Partial correlations between maximum movement reduction and average download speed in different municipalities using several attributes as control.

## 5 Mediation analysis

Mediation analysis is used to assess the direct and indirect effect of a regressor $X$ on the outcome $Y$, considering another regressor $M$ as a mediator [9]. The direct effect refers to the relationship between $X$ and $Y$, independent of $M$. Said differently, it is the effect of the independent variable on the outcome that is not explained by the mediator. On the contrary, the indirect effect refers to the relationship between the independent variable and the outcome that is explained by the mediator. In practice, mediation analysis works as follows. First, the regression of $X$ (but not $M$) on the outcome variable $Y$ is conducted. The coefficient of $X$ in this regression represents the total effect $T_X$. Second, the regression is repeated with $M$ included as a regressor. The coefficient obtained for $X$ in this regression represents the direct effect $D_X$. Finally, the indirect effect is calculated as the difference between the total and direct effects, that is $I_X = T_X - D_X$. In our case, we want to test whether, the impact of internet speed on NPIs compliance (measured by the maximum movement change observed in 2020) is fully mediated by RWI or whether there is a residual, significant direct effect. The statistical analysis is performed using the python package *pingouin* [8]. Besides the RWI as a mediator, we consider the other features introduced in the main text as normal regressors. In Tab. 5, we report the results of the mediation analysis for the three countries. The table reports i) the total effect, which represents the coefficient of the internet speed in a regression considering among regressors the internet speed itself and the other covariates but not the RWI (mediator), ii) the direct effect, which is instead the coefficient of the internet speed resulting from a regression considering also the RWI among regressors, and iii) the indirect effect which is the difference between the total and the direct effect and it represents the portion of the effect of the internet speed on NPIs compliance which is mediated by the RWI. We obtain a significant indirect effect only in the case of Colombia, while we obtain a direct effect in all three cases. This indicates that, according to this analysis, internet speed significantly and directly influences NPIs compliance, even tough this effect can be only partially mediated by the RWI.

| | **Colombia** | **Ecuador** | **El Salvador** |
|---|---|---|---|
| *Total Effect* | 0.53 [0.44 ; 0.63 | 0.14 [-0.02 ; 0.29] | 0.41 [0.15 ; 0.67] |
| *Direct Effect* | 0.30 [0.21 ; 0.39] | 0.16 [0.003 ; 0.32] | 0.35 [0.12 ; 0.57] |
| *Indirect Effect* | 0.23 [0.18 ; 0.32] | -0.02 [-0.08 ; 0.01] | 0.06 [-0.05 ; 0.18] |

Table 2: **Results of the mediation analysis**. We report total, direct, and indirect effects obtained from the mediation analysis considering the Relative Wealth Index as a mediator of the effect of internet speed on NPIs compliance.

# 6 Regularized linear models and bootstrapping

## 6.1 Ridge regression

Ridge regression is a regularized linear model for analyzing data suffering from multicollinearity [10]. Indeed, when the independent variables are highly correlated, the standard errors estimated via ordinary least squares may be affected. More in detail, parameters $\beta_j$ are estimated minimizing the following objective function:

$$\mathcal{L}(\alpha) = \sum_{i=1}^{N}(y_i - \sum_{j=1}^{M} x_{ij}\beta_j)^2 + \alpha \sum_{j=1}^{M} \beta_j^2 \tag{1}$$

Where $N$ is the number of samples and $M$ is the number of independent features. With respect to the typical loss function of ordinary least squares, $\mathcal{L}(\alpha)$ has an additional L2 regularization term which is the sum of the squares of the weights multiplied by a parameter $\alpha$. This parameter modulates the importance of the regularization term. When $\alpha = 0$ we find the typical expression of ordinary least squares loss function, for higher values instead the L2 regularization becomes more important. In this work, the regularization parameter $\alpha$ is calibrated via leave-one-out cross-validation exploring 30 log-spaced values between 0.1 and 10. We use the implementation of Ridge regression in the Python package *scikit-learn* [11].

## 6.2 Bootstrap sampling

The bootstrap method is a technique for the estimation of regression parameters and confidence intervals. This approach consists in iteratively resampling the dataset with replacement and performing the estimation on the resampled data. More formally, consider the matrix of the independent variables $X \in \mathbf{R}^{N\times M}$ and correspondent array of the dependent variable $Y \in \mathbf{R}^{N}$. At each iteration, we sample with replacement $N$ observations from the dataset, obtaining $X^{bootstrap} \in \mathbf{R}^{N\times M}$ and $Y^{bootstrap} \in \mathbf{R}^{N}$. Then, we use $X^{bootstrap}$ and $Y^{bootstrap}$ to estimate the regression parameters $\hat{\beta}_j$. We repeat this procedure for $T = 500$ times. In the end, we use the results of different iterations to compute median and 95% confidence intervals for the parameters. This is a general methodology that can be applied to any model, from ordinary least squares to Ridge regression.

## 6.3 Multiple variable regression with Ridge and bootstrap sampling

We repeat the regression presented in the main text in which we used the maximum reduction as the dependent variable and multiple features as independent variables. Instead of ordinary least squares, we use Ridge regression and we perform parameter estimation using the bootstrap method. In Fig. 4 we compare the parameters estimated with OLS (presented in the main text in Fig.5-B) and with Ridge regression. As we can see, parameters obtained with the two approaches are compatible, therefore the overall picture commented in the main text remains valid. We acknowledge wider confidence intervals for the variable *cases* for Colombia. As mentioned in the main text, the low importance of this variable may be because, at the maximum of mobility reductions, most of the municipalities reported few or no cases, while a small number reported a consistent number of cases. Because of the iterative resampling, the estimation of this parameter may be affected by this distribution. In Fig. 5 we show the convergence of parameters estimated via bootstrapping. We plot the estimated medians and 95% as a function of the bootstrap step. We see that 500 steps are enough for convergence.

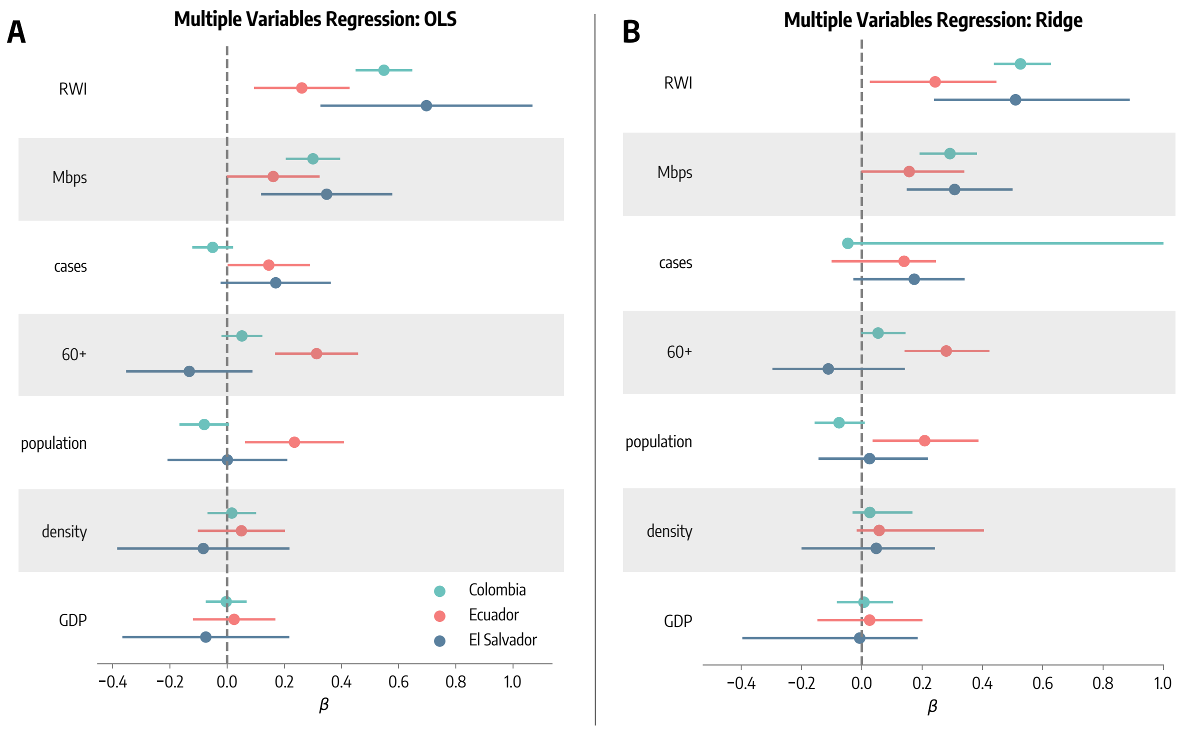


Figure 4: **Comparison of regression coefficients estimated with ordinary least squares and with Ridge regression and bootstrap sampling.**

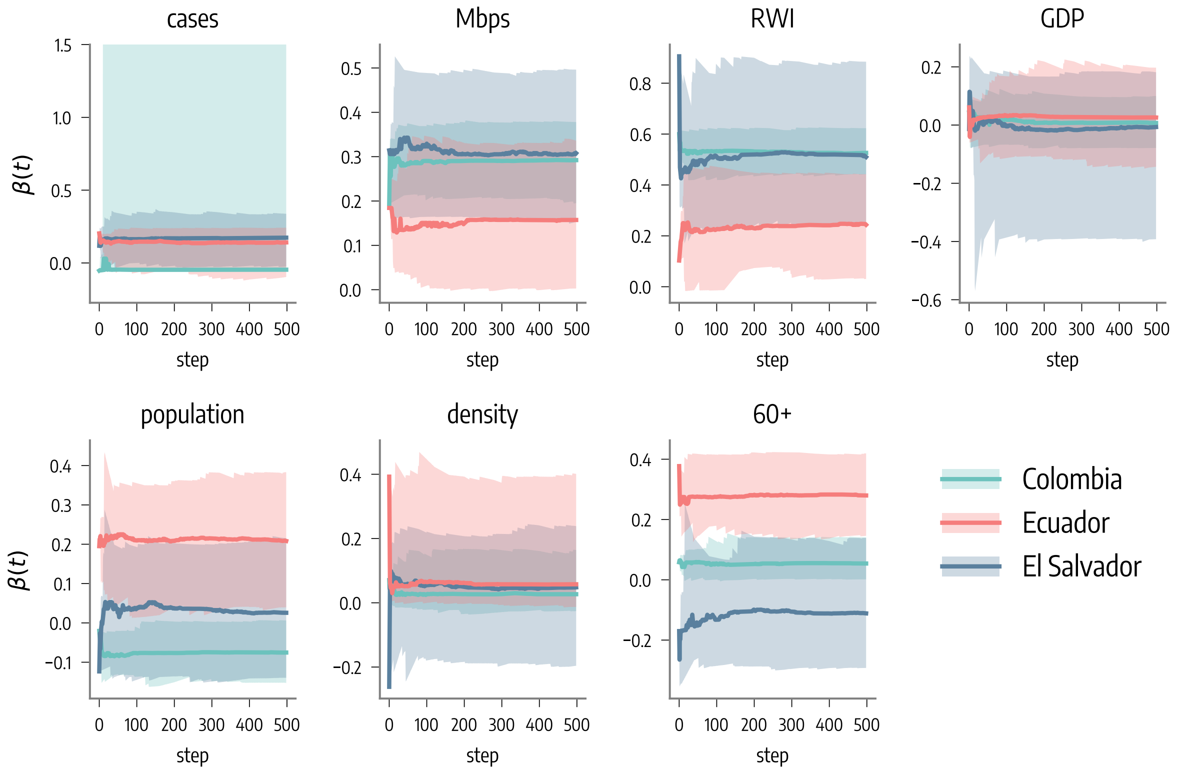


Figure 5: **Evolution of estimated median and 95% CI as a function of bootstrap step.**

# 7 The *Stay at Home* mobility metric

## 7.1 The metric

The *Movement Range Maps* dataset provides two main metrics, a percentage reduction in mobility with respect to a pre-pandemic baseline (*movement reduction*), and the percentage of users that appear to stay within a small area for the whole day (*stay at home*). This second metric is calculated by Meta by determining the percentage of users who are detected in only one $600m^2$ tile during a day. In the main text, we characterized the adherence to NPIs with the first metric, here we repeat the analyses using the second one.

In Fig. 6 we show the weekly evolution of the *stay at home* metric throughout 2020 for Colombia, Ecuador, and El Salvador (national averages as solid lines and minimum-maximum intervals as shaded areas), together with the Stringency Index from the Oxford COVID-19 Government Response Tracker [6]. We see that the metric has a similar trend of *movement reduction* presented in the main text. In March 2020 the fraction of people staying at home increased reaching a maximum in late March/early April (48% for Colombia, 53% for Ecuador, and 55% for El Salvador). After reaching the maximum, we observe an inversion, and the fraction of people remaining at home slowly starts to decrease. Also in this case we find that the mobility metric follows the evolution of the stringency index

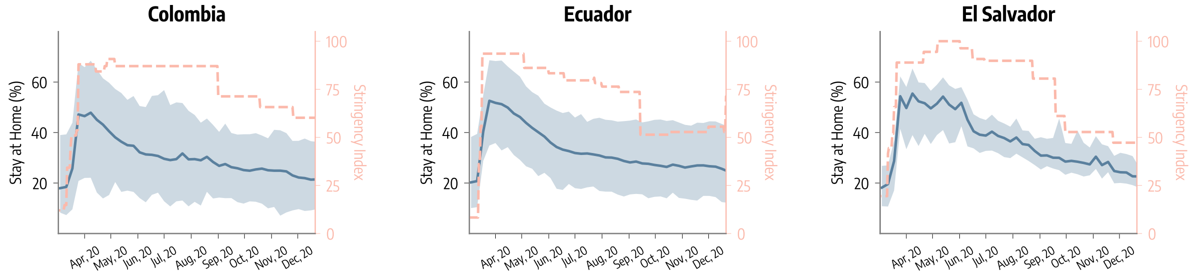


Figure 6: **Percentage of individuals staying home following the establishment of NPIs in Colombia, Ecuador, and El Salvador.** We show the *stay at home* metric between 2020/03/01 and 2020/12/31 for Colombia, Ecuador, and El Salvador. We show national average (solid line) and the minimum-maximum interval (shaded area) computed over all municipalities in the three countries for which we have data. We also show the stringency index (orange dashed line) of policies implemented to curb COVID-19 spread in the three countries.

## 7.2 Correlations

In Fig. 7-A we plot the average fixed download speed of municipalities against their maximum percentage of people staying at home during 2020. We find for all three countries positive and significant correlations (0.54 [0.47 – 0.61], for Colombia, 0.47 [0.33 – 0.58] for Ecuador, and 0.58 [0.36 – 0.74] for El Salvador). Furthermore, these coefficients remain significant also after controlling for the socioeconomic status of municipalities. Indeed, we find the following partial correlations using the Relative Wealth Index as a control: 0.27 [0.17 – 0.36], 0.43 [0.30 – 0.56], and 0.37 [0.10 – 0.60] for, respectively, Colombia, Ecuador, and El Salvador.

In Fig. 7-B we show the evolution of the Pearson correlation coefficient between the average download speed and the weekly fraction of people staying home during 2020. Also in this case we find that the correlation follows the evolution of the stringency index (orange dashed line in the figure).

## 7.3 Regressions

In Fig. 8 we show results of the static regression analysis using the maximum fraction of individuals staying at home as the dependent variable.

Fig. 8-A shows the results of the single variable regression. We find very similar results to those of the main text. Higher density and population are associated with stronger adherence to NPIs. Socioeconomic features are also important, indeed the coefficient of the $RWI$ is positive and significant in all three countries while the $GDP$ is significant only for El Salvador. A higher number of reported cases is

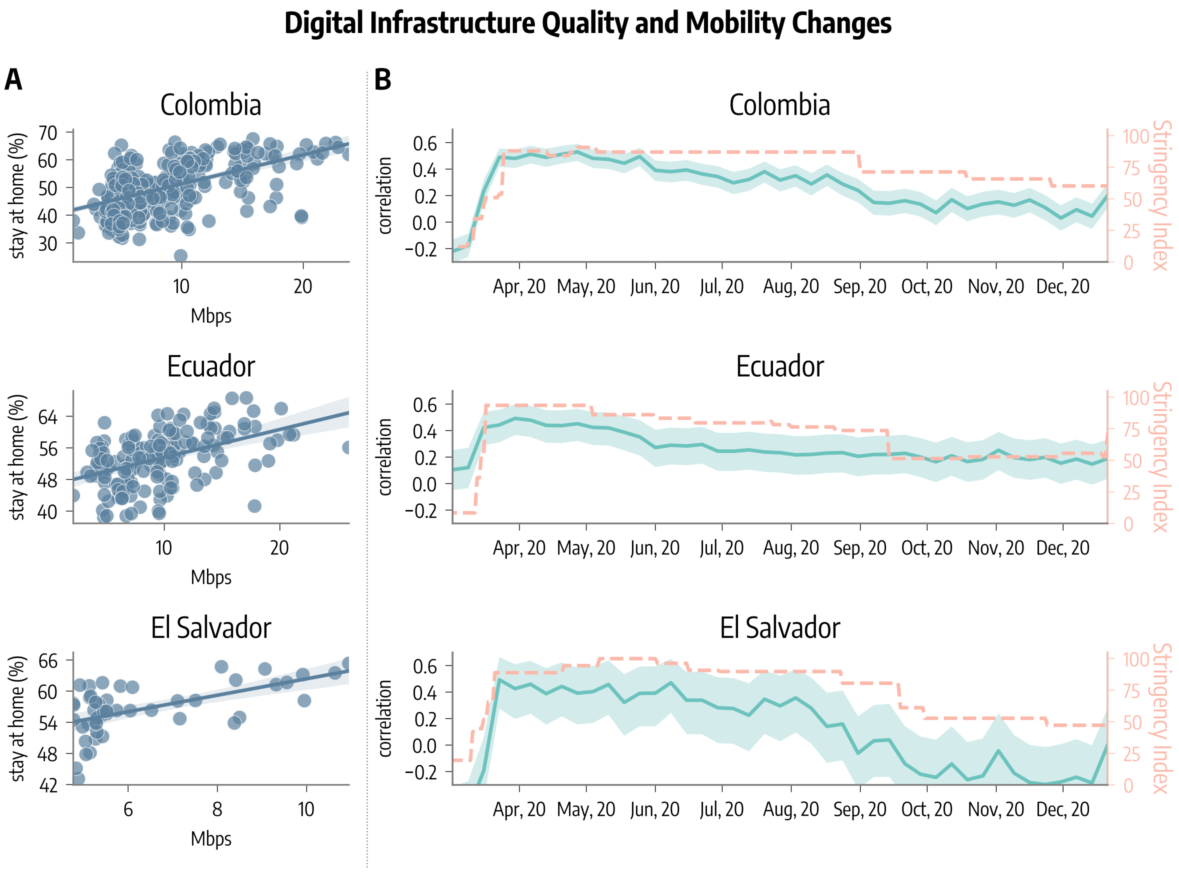


Figure 7: **Association between fraction staying at home and digital infrastructure quality in different municipalities of Colombia, Ecuador, and El Salvador.** A) We plot the greatest fraction of people staying at home against the average download speed in different municipalities. B) We plot the Pearson correlation coefficient (median and 95% CI) between average weekly *stay at home* metric and average download speed of different municipalities. The orange dashed lines in all plots represent the Stringency Index.

significantly associated with higher NPIs compliance in Ecuador. Finally, as expected from the correlation analysis, the association between the percentage of people staying home and digital infrastructure quality is positive and significant in all three countries. The role of average download speed is confirmed also in Fig. 8-B, where the results of the multiple variables regression are reported. Indeed, the coefficient of this variable remains positive and significant. Overall, we get wide confidence intervals. This may be due to multicollinearity between independent features. Indeed, in Fig. 8-C we repeat the multiple regression analysis using Ridge regression and bootstrapping for estimation. As expected, we obtain smaller confidence intervals. Population size and density remain significant predictors of NPIs adherence. The number of cases is significant only in the case of Ecuador and marginally significant for El Salvador. Interestingly, the role of socioeconomic figures is less clear. Indeed, we get that the RWI is positive and significant only in the case of Colombia, while the GDP is marginally significant only for El Salvador.

Finally, in Fig. 9 we show the results of the time-varying regression approach using the *stay at home* as dependent variable. The findings of the main text are confirmed. Indeed, across the three countries the coefficient of internet speed is $\sim 0$ at the beginning of March and then grows reaching a peak in late March/April, concurrently with the maximum strictness of restrictive policies. Also in this case, the $wMAPE$ tends to be lower when the NPIs in place are stricter (i.e., higher values of the Stringency Index). The third row confirms the importance of the variable $Mbps$, especially during the early months of 2020, where we obtain $\Delta_{AIC}$ values that are smaller than $-2$.

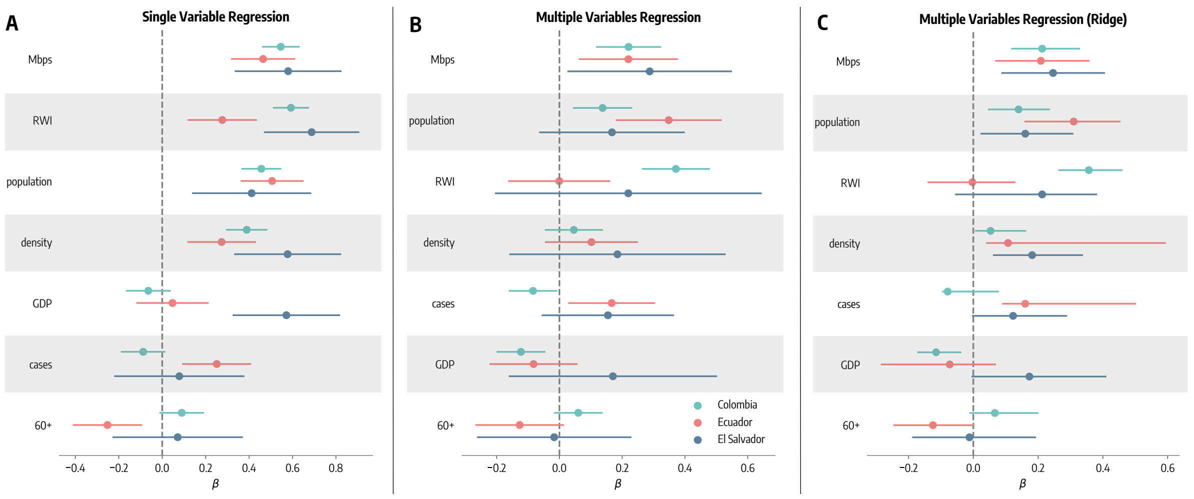


Figure 8: **Coefficients of the regressions (maximum *stay at home* as dependent variable).** A) Coefficients of the single variable regression. B) Coefficients of the multiple variables regression. C) Coefficients of the multiple variables regression using Ridge and bootstrapping for estimation.

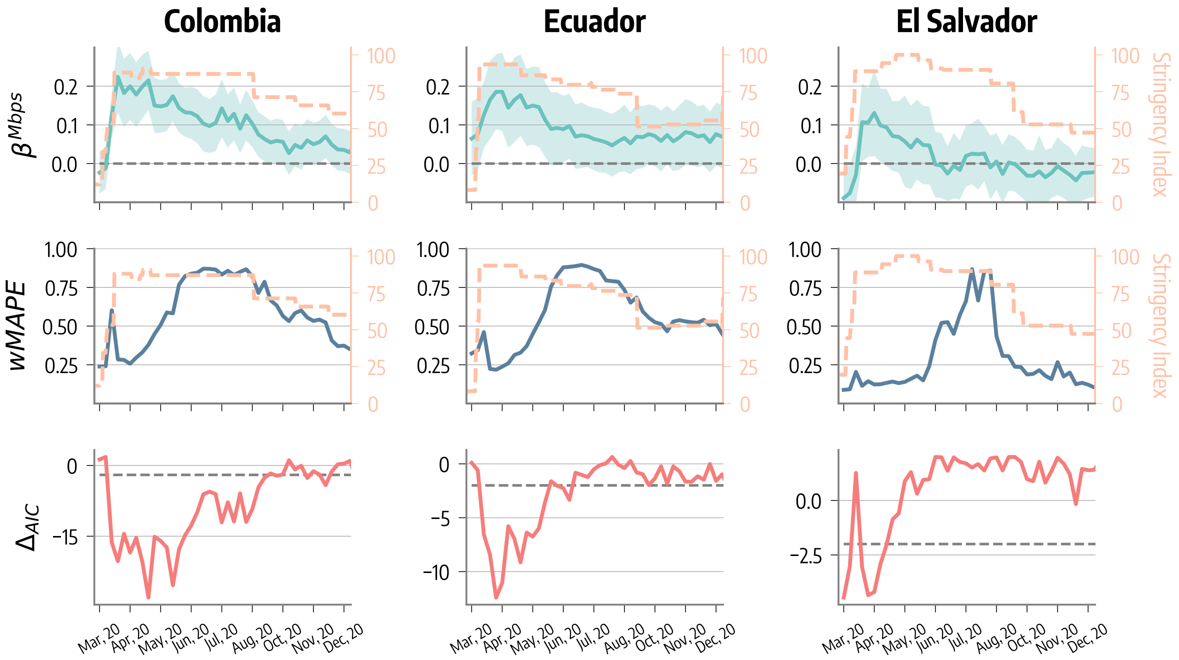


Figure 9: **Weekly regression results (Stay home as dependent variable).** First line shows the evolution of the regression coefficient of the average download speed as a function of time for the three countries. Second line shows the weighted mean absolute percentage error $wMAPE$ in different weeks. Third line shows the evolution of the $\Delta_{AIC}$ between the model with and without download speed as an independent variable. We average all quantities over a month to rule out the influence of noise. The orange dashed line in the different figures represents the evolution of the Stringency Index.

# 8 Digital tools adoption and NPIs adherence

## 8.1 Internet adoption and infrastructure quality

In the main text, we considered the quality of digital infrastructure as a possible determinant of NPIs adherence. Arguably, also the actual adoption of digital tools is an important factor playing a role in NPIs adherence. Access to connectivity significantly increased over the past years in Latin America [12]. This is reflected, for example, in the positive trend observed for the number of fixed internet subscriptions per 100 people [13] shown in Fig. 10-A. Despite this encouraging tendency, the gap with High-Income countries is still worryingly wide. In Colombia and Ecuador, the number of fixed internet subscriptions is comparable to that of other countries in the same region while in El Salvador it is significantly lower. If we push our analysis to a more granular level than general national trends we uncover an additional layer of digital inequality. As an illustrative example, we use data on fixed internet subscriptions per 100 (2019) in the different departments of Colombia [14]. The national average is 14.1 subscriptions per 100 but this number alone does not communicate the vast heterogeneity across regions. Indeed, as shown in Fig. 10-B, the internet penetration widely varies across departments, from a maximum of 25.2 fixed subscriptions per 100 in the Bogota Department to a minimum of 0.22 in the Vaupes Department. Finally, we mention that internet adoption and quality of connectivity are not unrelated. For example, we find a strong significant correlation between the number of fixed internet subscriptions per 100 and the related average download speed measured with Ookla® Speedtest Intelligence® data in the municipalities of Colombia ($\rho = 0.63$, 95% CI: $[0.58 - 0.69]$).

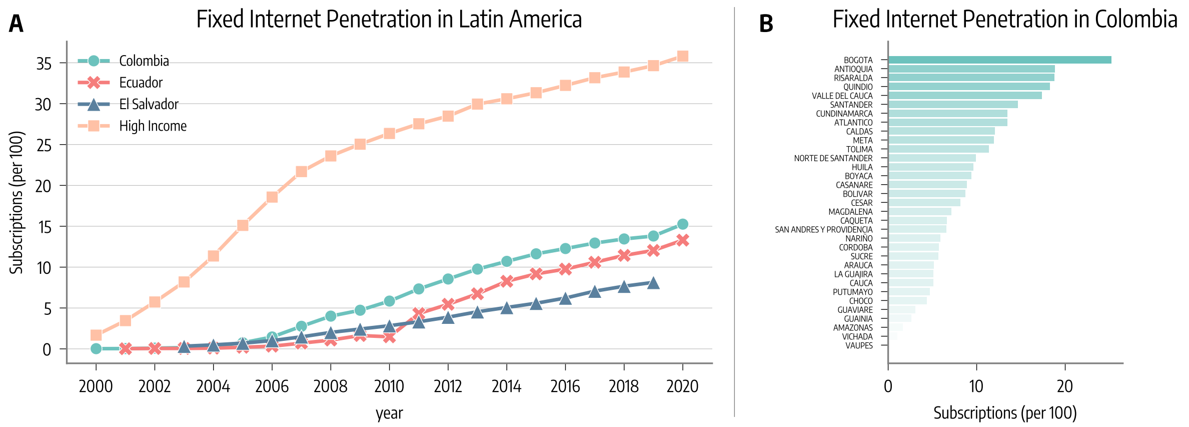


Figure 10: A) Number of fixed internet subscriptions per 100 in high-income countries, Colombia, Ecuador, and El Salvador. B) Number of fixed internet subscriptions per 100 in Colombian departments.

## 8.2 Proxies of digital tools adoption

As the first proxy for internet adoption, we use the number of fixed internet subscriptions per 100. To the best of our knowledge, this information is only available at the municipal level for Colombia from Ref. [14]. As a second proxy, we consider the number of unique devices per 100 that performed a Speedtest® during 2019-2020. This is more than just a measure of internet adoption. Indeed, among internet users only the more digitally aware know about these types of services. We compute it for the three countries using the Ookla® dataset that we used for the calculation of average download speed.

## 8.3 Correlations between digital tools adoption and NPIs adherence

In Fig. 11 we plot the number of tests performed per 100 against NPIs adherence (described by either the *movement reduction* or the *stay at home* metric). To reduce the influence of outliers in the number of tests per capita, we take the logarithm of this quantity. Overall, we find that a higher number of tests is associated with higher compliance. Indeed, the Pearson correlation coefficients reported in the figure vary from a minimum of 0.24 to a maximum of 0.61. In Tab. 3 we report the relative partial correlations

using the relative wealth index as a control. As expected, the coefficients are smaller, but still significant in most of the cases. Only for El Salvador, we get non-significant or marginally significant coefficients.

Finally, we repeat the analysis considering the number of fixed internet subscriptions per 100 rather than the number of tests performed. As explained before, we have this information only for the municipalities of Colombia. We obtain a highly significant correlation coefficient between internet penetration and both maximum movement reduction (0.65, [0.59; 0.71]) and the maximum fraction of individuals staying at home (0.68 [0.62; 0.73]). The correlations remain significant also after controlling for the socioeconomic status of different municipalities using the relative wealth index (partial correlation of, respectively, 0.29 [0.20; 0.38] and 0.45 [0.37; 0.52]).

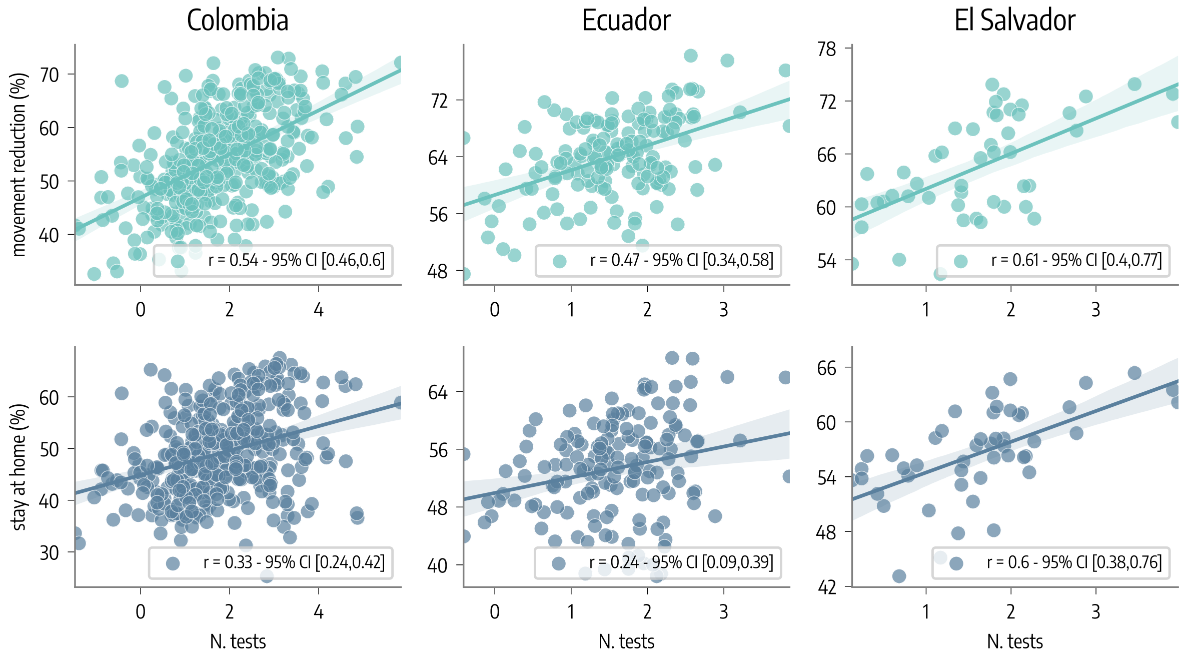


Figure 11: Scatter plot between number of Speedtest® measurement and NPIs adherence (described by either the *movement reduction* and the *stay at home* metric).

| | Movement Reduction | Stay at Home |
|---|---|---|
| *Colombia* | 0.36 [0.27; 0.44] | 0.10 [0.0; 0.2] |
| *Ecuador* | 0.40 [0.26; 0.52] | 0.18 [0.02; 0.33] |
| *El Salvador* | 0.20 [-0.09; 0.46] | 0.25 [-0.04; 0.5] |

Table 3: Partial Pearson correlation coefficients between NPIs adherence and number of tests per 100 using the relative wealth index as control.

## 8.4 Regression analysis

We repeat the static regression in which we regress the maximum adherence to NPIs (either maximum *movement reduction* or *stay at home*) against several independent features including the proxies of adoption of digital tools just described.

Before moving to the regression, we estimate the correlations between independent features in Fig. 12. The number of tests performed is generally correlated with the quality of the digital infrastructure (0.50, 0.36, and 0.51 in the municipalities of Colombia, Ecuador, and El Salvador). At the same time is also correlated with the socioeconomic status of the municipalities. Indeed, the correlation between the number of tests performed and the relative wealth index is, in the three countries, 0.43, 0.31, and

0.68. In El Salvador, it is also correlated with GDP per capita (0.71). The number of fixed internet subscriptions in Colombia is also positively associated with higher download speed (0.64) and higher socioeconomic status (0.72 with relative wealth index). The maximum variance inflation factors are, respectively, 2.8, 1.6, and 5.7 for Colombia, Ecuador, and El Salvador. Because of these values, we will use Ridge regression and bootstrapping for the estimation of coefficients.

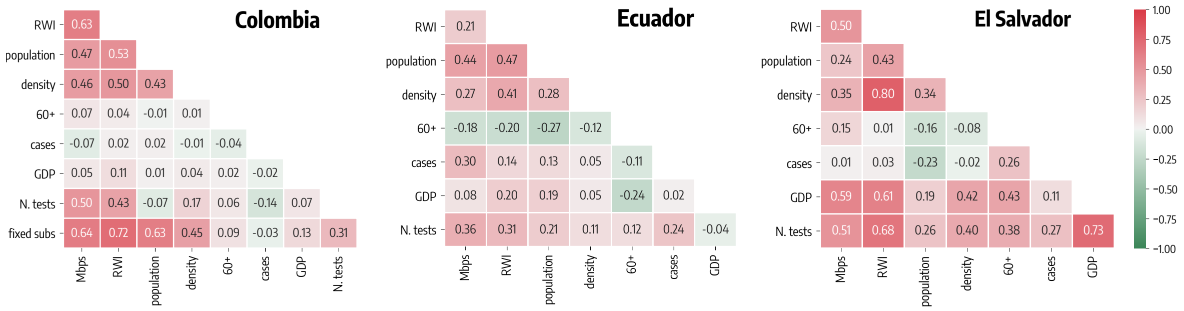


Figure 12: **Correlations between independent features (proxies of adoption of digital tools included)**

In Fig. 13 we report the coefficients of the static regression with the additional features on digital tools adoption included. When the dependent variable is the maximum *movement reduction*, we obtain that the digital infrastructure quality is still a significant predictor for Colombia and El Salvador. In the case of Ecuador, the coefficient is still positive but not significant. We notice that the coefficient of the number of fixed tests performed per 100 is positive and significant in all three countries. The important role of digital tools adoption is confirmed by the coefficient of the number of fixed internet subscriptions per 100 in Colombia. We also estimate a model considering only the number of fixed tests performed per 100 and not internet speed. We obtain an $R^2$ of 0.58, 0.38, and 0.64 for Colombia, Ecuador, and El Salvador, and the following coefficients for number of tests: 0.32 ([0.24, 0.40]), 0.28 ([0.13, 0.42]), and 0.23 ([0.12, 0.59]).

The picture emerging using the maximum *stay at home* as the dependent variable is consistent. The coefficient of the number of Speedtest® measurements is marginally significant for Colombia and El Salvador, while the average download speed remains a significant predictor in the case of Ecuador and El Salvador. We still obtain a highly positive and significant coefficient for the number of fixed internet subscriptions in Colombia.

Overall, the analysis presented here shows the possible importance of digital tools adoption and confirms the results presented in the main text.

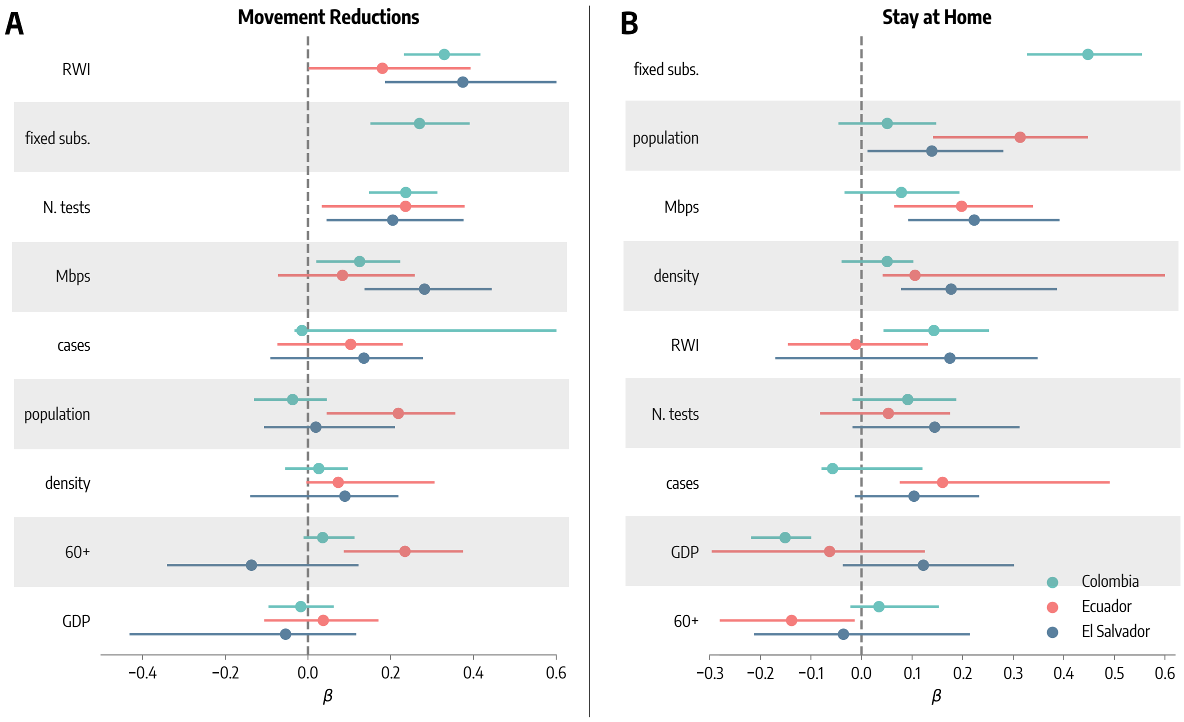


Figure 13: **Coefficients of the regressions (proxies of adoption of digital tools included).**

# 9 Formal employment and labor structure

We include in our regression data about employment. To the best of our knowledge, this data are available at the municipal level only Colombia, therefore we will restrict our analysis to this country only. First, we consider labor formality. This is simply computed dividing the number of individuals formally employed by the number of people in working age $(15-64)$ in a given municipality [15, 16]. Employment data are provided in Ref. [17], while subnational population statistics in Ref. [18]. Second, we consider the relative importance of different economic sectors. From Ref. [19] we get the GDP of primary, secondary, and tertiary sector. Then, we compute the share of the total municipal GDP that corresponds to each sector. In the regression we include only the share of GDP associated with primary and tertiary sectors. Indeed, the third variable (i.e., the share of the secondary sector) would be redundant since the sum of the three shares is always 1.

We repeat the multiple variable regression including these additional variables. Coefficients are reported in Fig. 14. We notice that, even after including these additional features, the coefficients associated with digital infrastructure (*Mbps*) and digital tools adoption (*N. tests* and *fixed subs*) are still positive and significant. The only exception is the number of consumer-initiated tests taken with Speedtest® performed, whose coefficient is not significant and close to 0 in the case of the *stay at home* metric as dependent feature. Labor formality and the share of GDP associated with the tertiary sector is not significant in both cases. The coefficient of the share of GDP associated with the primary sector is instead negative and significant for movement reductions. This is in line with what expected, indeed the primary sector is mainly associated with essential activities that must continue also during lockdowns and that can hardly be performed remotely.

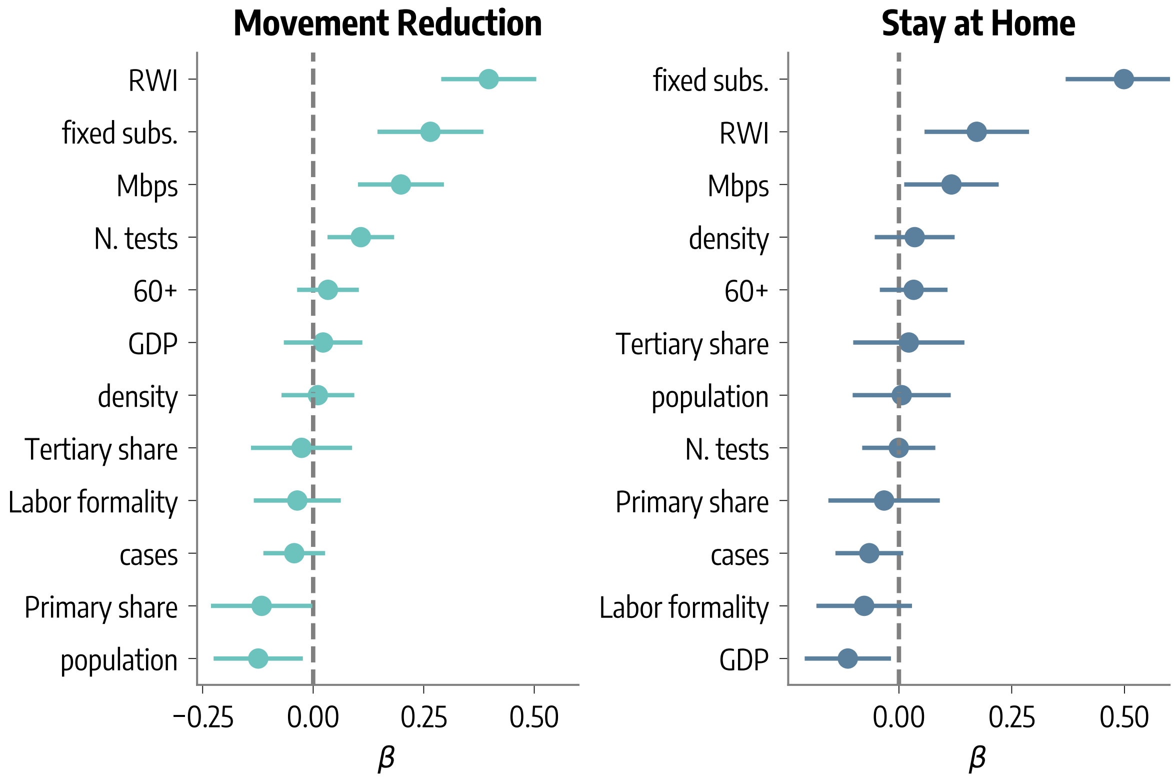


Figure 14: **Coefficients of the regressions for Colombia (employment data and importance of economic sectors included).**

## References


[1] Medida de pobreza multidimensional municipal de fuente censal 2018. https://www.dane.gov.co/index.php/estadisticas-por-tema/pobreza-y-condiciones-de-vida/pobreza-y-desigualdad/medida-de-pobreza-multidimensional-de-fuente-censal, 2021. Accessed: 2021-09-07.

[2] J. Illingworth and F. Campaña. *Informe sobre Desarrollo Humano del Ecuador*. Fundación Ecuador, 2019.

[3] Almanaque 262. Estado del desarrollo humano en los municipios de El Salvador. https://www.sv.undp.org/content/el_salvador/es/home/library/hiv_aids/almanaque-262.html, 2021. Accessed: 2021-09-07.

[4] Sabina Alkire and Maria Emma Santos. Measuring acute poverty in the developing world: Robustness and scope of the multidimensional poverty index. *World Development*, 59:251–274, 2014.

[5] Mark McGillivray and Howard White. Measuring development? The UNDP's human development index. *Journal of international development*, 5(2):183–192, 1993.

[6] Thomas Hale, Noam Angrist, Rafael Goldszmidt, Beatriz Kira, Anna Petherick, Toby Phillips, Samuel Webster, Emily Cameron-Blake, Laura Hallas, Saptarshi Majumdar, and Helen Tatlow. A global panel database of pandemic policies (Oxford COVID-19 Government Response Tracker). *Nature Human Behaviour*, 5(4):529–538, 2021.

[7] Richard Arnold Johnson and Dean W. Wichern. *Applied multivariate statistical analysis*. Prentice Hall, 5. ed edition, 2002.

[8] Raphael Vallat. Pingouin: statistics in python. *Journal of Open Source Software*, 3(31):1026, 2018.

[9] Andrew F Hayes and Nicholas J Rockwood. Regression-based statistical mediation and moderation analysis in clinical research: Observations, recommendations, and implementation. *Behaviour research and therapy*, 98:39–57, 2017.

[10] Arthur E. Hoerl and Robert W. Kennard. Ridge regression: Biased estimation for nonorthogonal problems. *Technometrics*, 42(1):80–86, 2000.

[11] F. Pedregosa, G. Varoquaux, A. Gramfort, V. Michel, B. Thirion, O. Grisel, M. Blondel, P. Prettenhofer, R. Weiss, V. Dubourg, J. Vanderplas, A. Passos, D. Cournapeau, M. Brucher, M. Perrot, and E. Duchesnay. Scikit-learn: Machine learning in Python. *Journal of Machine Learning Research*, 12:2825–2830, 2011.

[12] Individuals using the Internet (% of population) - Latin America & Caribbean. https://data.worldbank.org/indicator/IT.NET.USER.ZS?locations=ZJ. Accessed: 2021-09-06.

[13] The World Bank, Fixed Broadband Subscriptions. https://data.worldbank.org/indicator/IT.NET.BBND, 2021. Accessed: 2021-11-08.

[14] Colombia - Fixed Internet Penetration at the Municipal Level. https://www.datos.gov.co/Ciencia-Tecnolog-a-e-Innovaci-n/Internet-Fijo-Penetraci-n-Municipio/fut2-keu8, 2021. Accessed: 2021-11-08.

[15] Neave O'Clery, Andres Gomez-Lievano, and Eduardo Lora. The path to labor formality: urban agglomeration and the emergence of complex industries. *CID Research Fellow and Graduate Student Working Paper Series*, 2016.

[16] Samuel Heroy, Isabella Loaiza, Alex Pentland, and Neave O'Clery. Covid-19 policy analysis: labour structure dictates lockdown mobility behaviour. *Journal of The Royal Society Interface*, 18(176):20201035, 2021.

[17] El Atlas Colombiano de Complejidad Económica. http://datlascolombia.com/#/downloads, 2021. Accessed: 2022-01-04.

[18] Colombia - Subnational Population Statistics. https://data.humdata.org/dataset/colombia-population-estimates-and-projections, 2021. Accessed: 2022-01-04.

[19] National Administrative Department of Statistics of Colombia, Cuentas nacionales departamentales: PIB por departamento. https://www.dane.gov.co/index.php/estadisticas-por-tema/cuentas-nacionales/cuentas-nacionales-departamentales, 2021. Accessed: 2022-01-04.
